# Supplementary material for: Status of Cassava Witches’ Broom Disease in the Philippines and Identification of Potential Pathogens by Metagenomic Analysis
Source: Biology (Basel). 2024 Jul 15;13(7):522. doi: 10.3390/biology13070522 (PMC11273669; doi:10.3390/biology13070522)
Supplement: Supplementary file 1 [file biology-13-00522-s001.zip › Figure S1-CWBD symptom rating scale.pdf]

Figure S1. CWBD symptom rating scale[1]

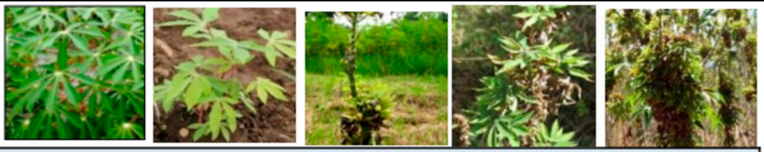

RATING	CLASSIFICATION	DESCRIPTION
1	No Infection	No symptoms observed and plants appear healthy.
3	Light Infection	Onset of mild chlorosis or anthocyanescence on the apical portion of the plant mainly on the younger leaves. The leaves become stiff, but most appear healthy.
5	Moderate Infection	Pronounced symptoms of witches broom, excessive anthocyanin pigmentation or yellowing of leaves, stiff leaves, shortening of the internodes and onset of multiple axillary bud growth are observed.
7	Severe Infection	Advanced symptoms of infection including yellowing of leaves , extreme shortening of internodes, bunchy top, profuse auxiliary bud formation and distortion of leaf deformation
9	Very Severe Infection	There is severe leaf reduction of most leaves accompanied by severe stunting of plants profuse auxiliary bud production and excessive branching, browning of mature leaves.

[1]Provided by the Bureau of Plant Industry Crop Pest Management Division under the National Survey and Early-Warning on Cassava Pests and Diseases project
