# Supplementary material for: Status of Cassava Witches’ Broom Disease in the Philippines and Identification of Potential Pathogens by Metagenomic Analysis
Source: Biology (Basel). 2024 Jul 15;13(7):522. doi: 10.3390/biology13070522 (PMC11273669; doi:10.3390/biology13070522)
Supplement: Supplementary file 1 [file biology-13-00522-s001.zip › Figure S3-Phylogenetic tree of phytoplasma and other bacteria.pdf]

Figure S3. Phylogenetic tree of phytoplasma and other bacteria

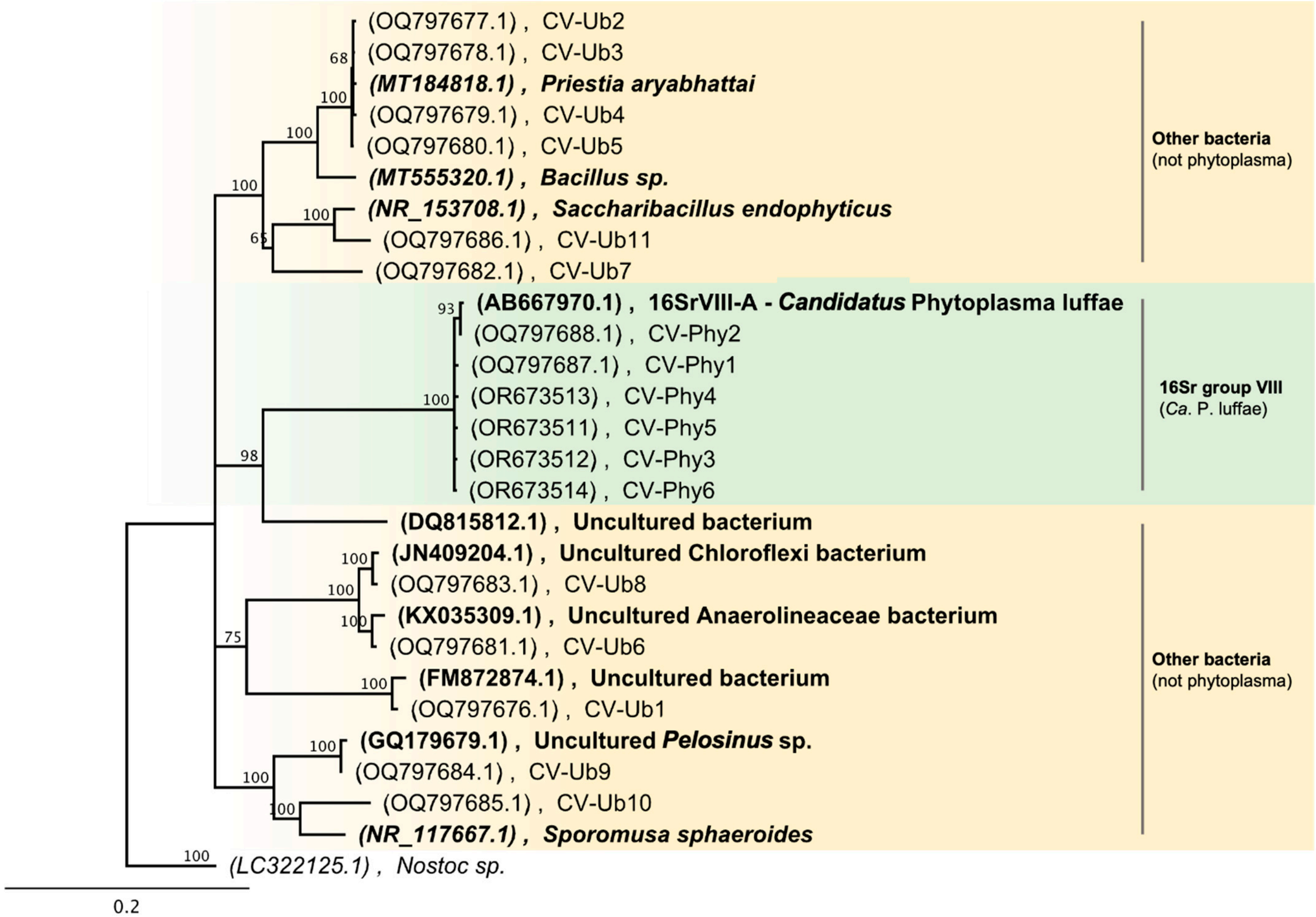

Phylogenetic tree based on the 16S rRNA gene sequences from CWBD symptomatic cassava. Constructed using Neighbor-Joining (NJ) tree build method. Sequences in this study were compared with phytoplasma strains and other uncultured bacteria (in bold label). Nostoc sp. (LC322125.1) was used as an outgroup. The scale bar indicates 0.2 substitutions per site. Accession numbers are enclosed in parentheses.
