# Supplementary material for: Status of Cassava Witches’ Broom Disease in the Philippines and Identification of Potential Pathogens by Metagenomic Analysis
Source: Biology (Basel). 2024 Jul 15;13(7):522. doi: 10.3390/biology13070522 (PMC11273669; doi:10.3390/biology13070522)
Supplement: Supplementary file 1 [file biology-13-00522-s001.zip › Figure S4-Bacterial community composition of unfiltered amplicon sequencing reads.pdf]

Figure S4. Bacterial community composition of unfiltered amplicon sequencing reads

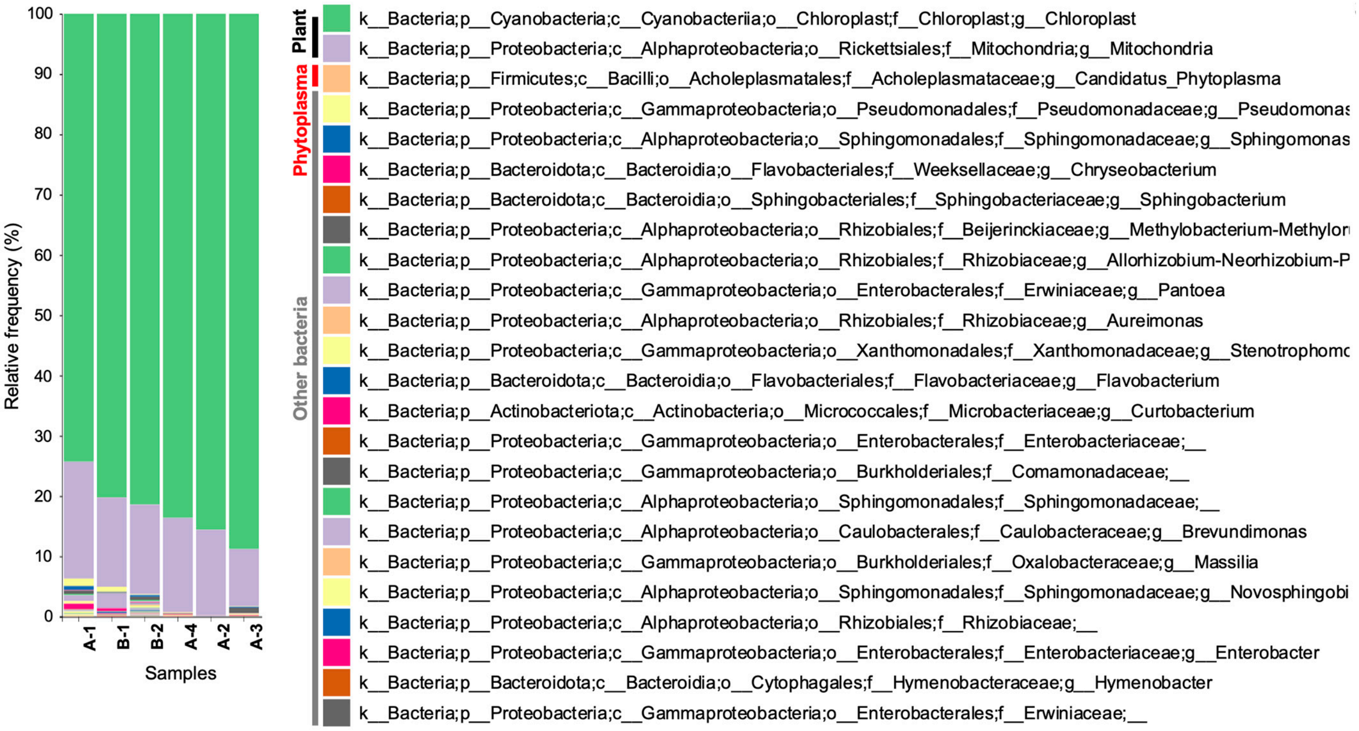

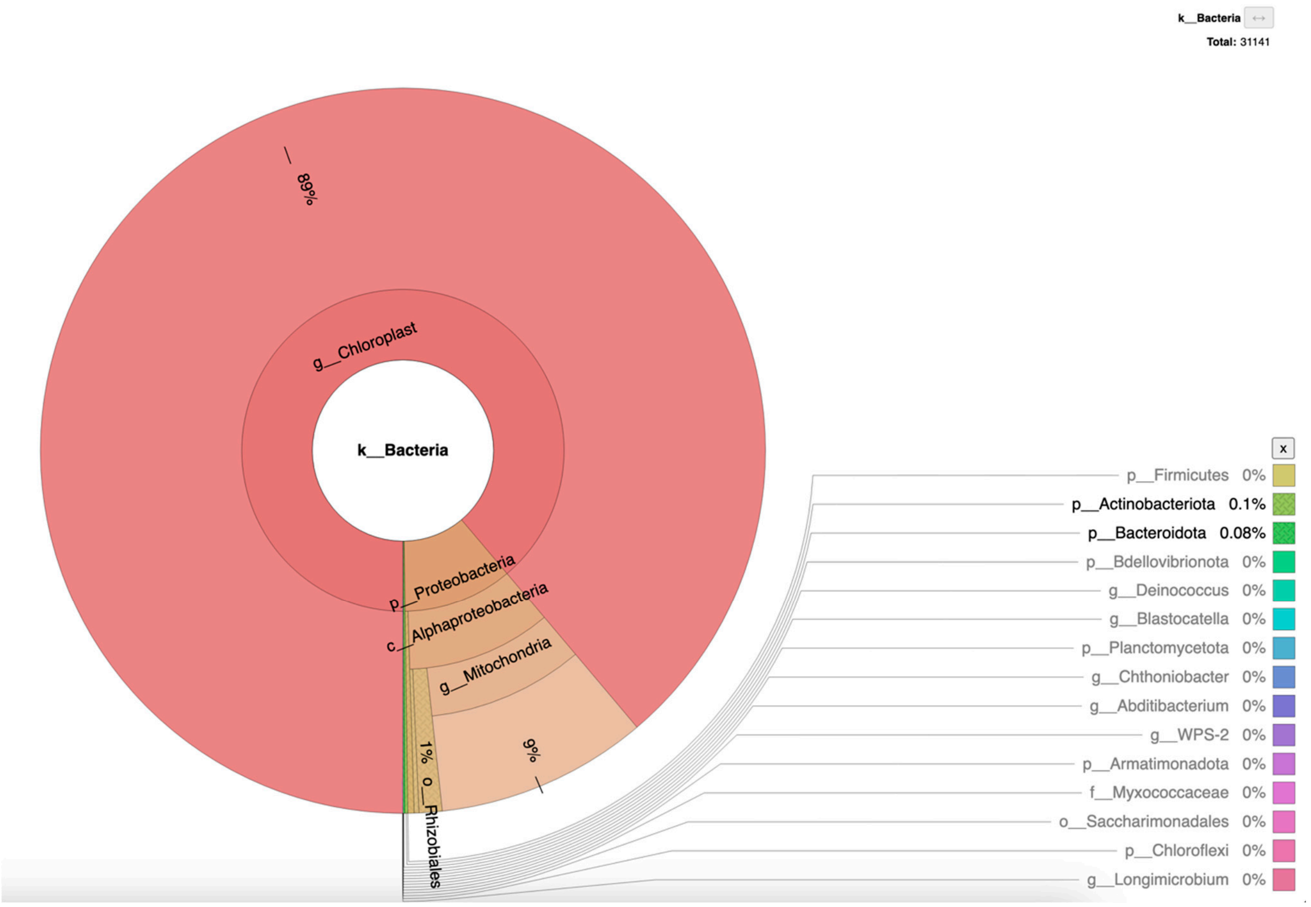

A-1

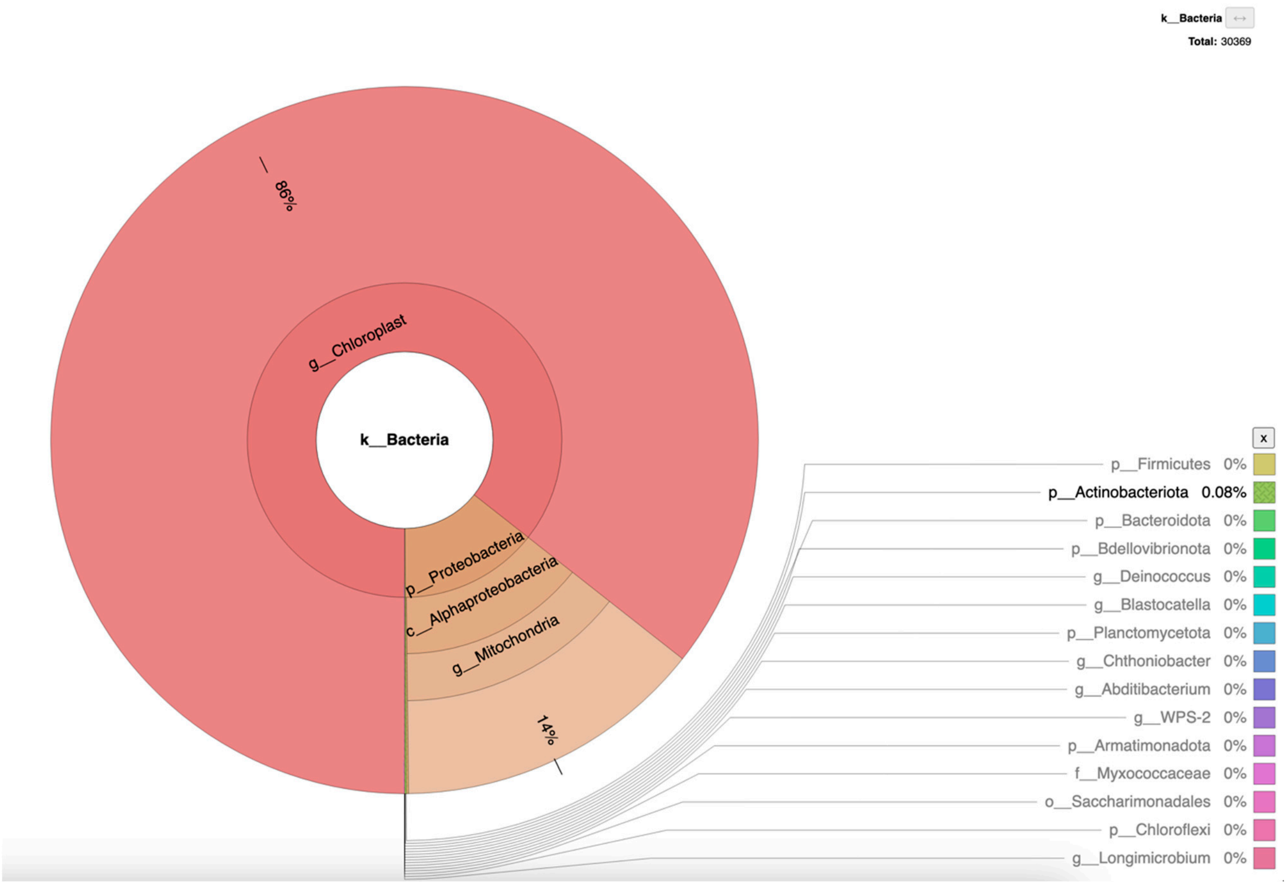

k__Bacteria
Total: 30369
k__Bacteria
g__Chloroplast
86%
p__Proteobacteria
c__Alphaproteobacteria
g__Mitochondria
14%
p__Firmicutes 0%
p__Actinobacteriota 0.08%
p__Bacteroidota 0%
p__Bdellovibrionota 0%
g__Deinococcus 0%
g__Blastocatella 0%
p__Planctomycetota 0%
g__Chthoniobacter 0%
g__Abditibacterium 0%
g__WPS-2 0%
p__Armatimonadota 0%
f__Myxococcaceae 0%
o__Saccharimonadales 0%
p__Chloroflexi 0%
g__Longimicrobium 0%

A-2

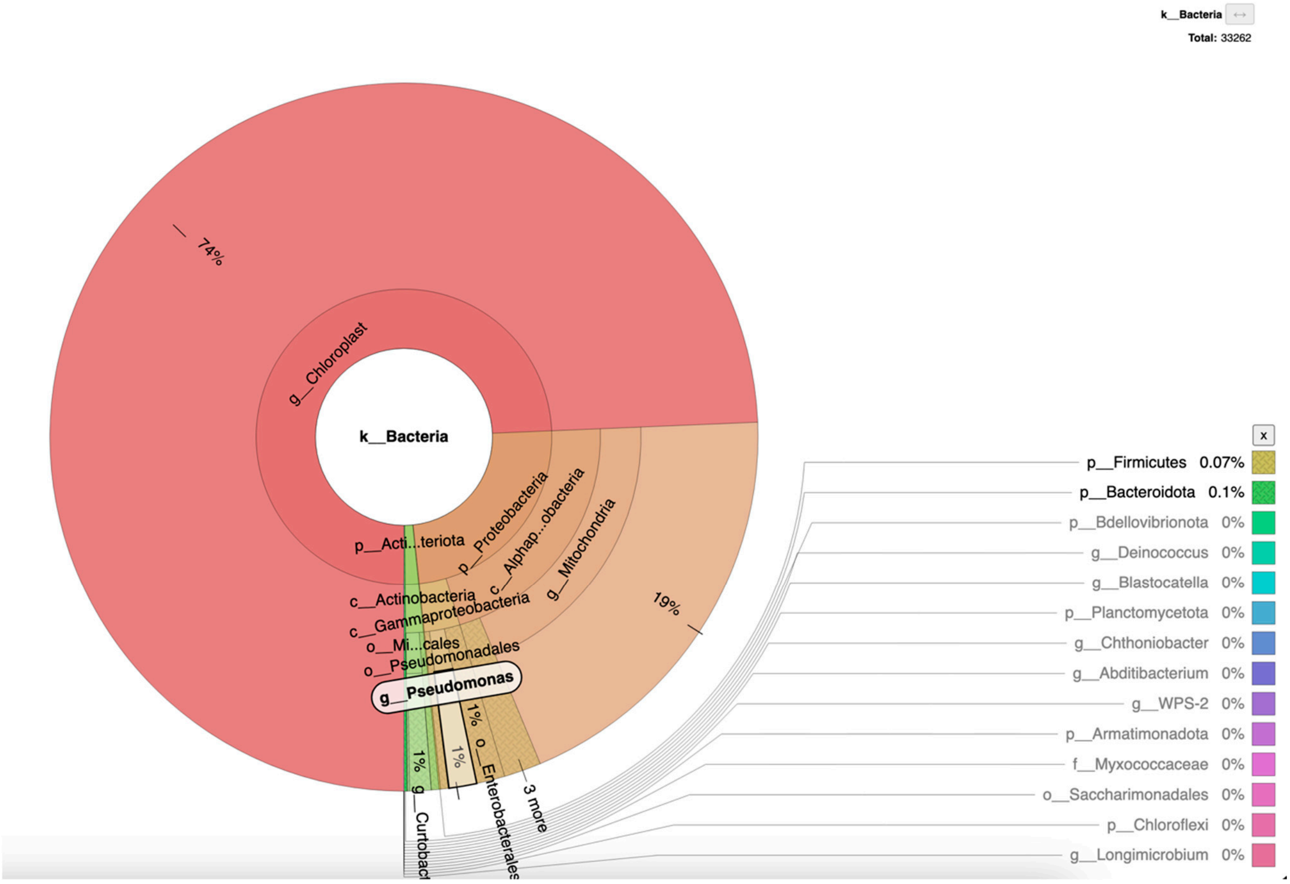

k__Bacteria
Total: 33262
k__Bacteria
g__Chloroplast
74%
p__Acti...teriota
p__Proteobacteria
c__Alphap...obacteria
g__Mitochondria
19%
c__Actinobacteria
c__Gammaproteobacteria
o__Mi...cales
o__Pseudomonadales
g__Pseudomonas
1%
1%
1%
o__Enterobacterales
3 more
p__Firmicutes 0.07%
p__Bacteroidota 0.1%
p__Bdellovibrionota 0%
g__Deinococcus 0%
g__Blastocatella 0%
p__Planctomycetota 0%
g__Chthoniobacter 0%
g__Abditibacterium 0%
g__WPS-2 0%
p__Armatimonadota 0%
f__Myxococcaceae 0%
o__Saccharimonadales 0%
p__Chloroflexi 0%
g__Longimicrobium 0%

A-3

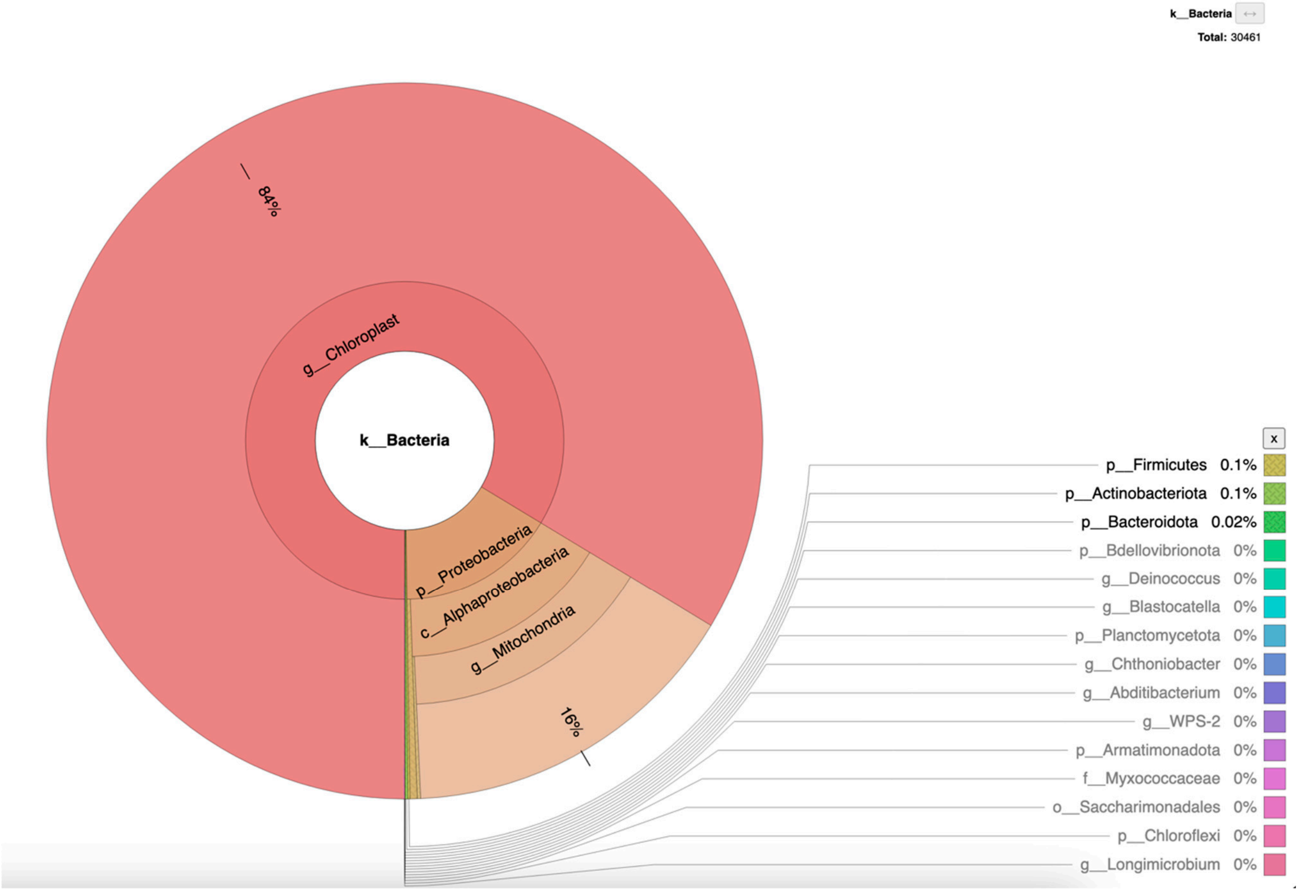
k__Bacteria
Total: 30461
84%
g__Chloroplast
k__Bacteria
p__Proteobacteria
c__Alphaproteobacteria
g__Mitochondria
16%
p__Firmicutes 0.1%
p__Actinobacteriota 0.1%
p__Bacteroidota 0.02%
p__Bdellovibrionota 0%
g__Deinococcus 0%
g__Blastocatella 0%
p__Planctomycetota 0%
g__Chthoniobacter 0%
g__Abditibacterium 0%
g__WPS-2 0%
p__Armatimonadota 0%
f__Myxococcaceae 0%
o__Saccharimonadales 0%
p__Chloroflexi 0%
g__Longimicrobium 0%

A-4

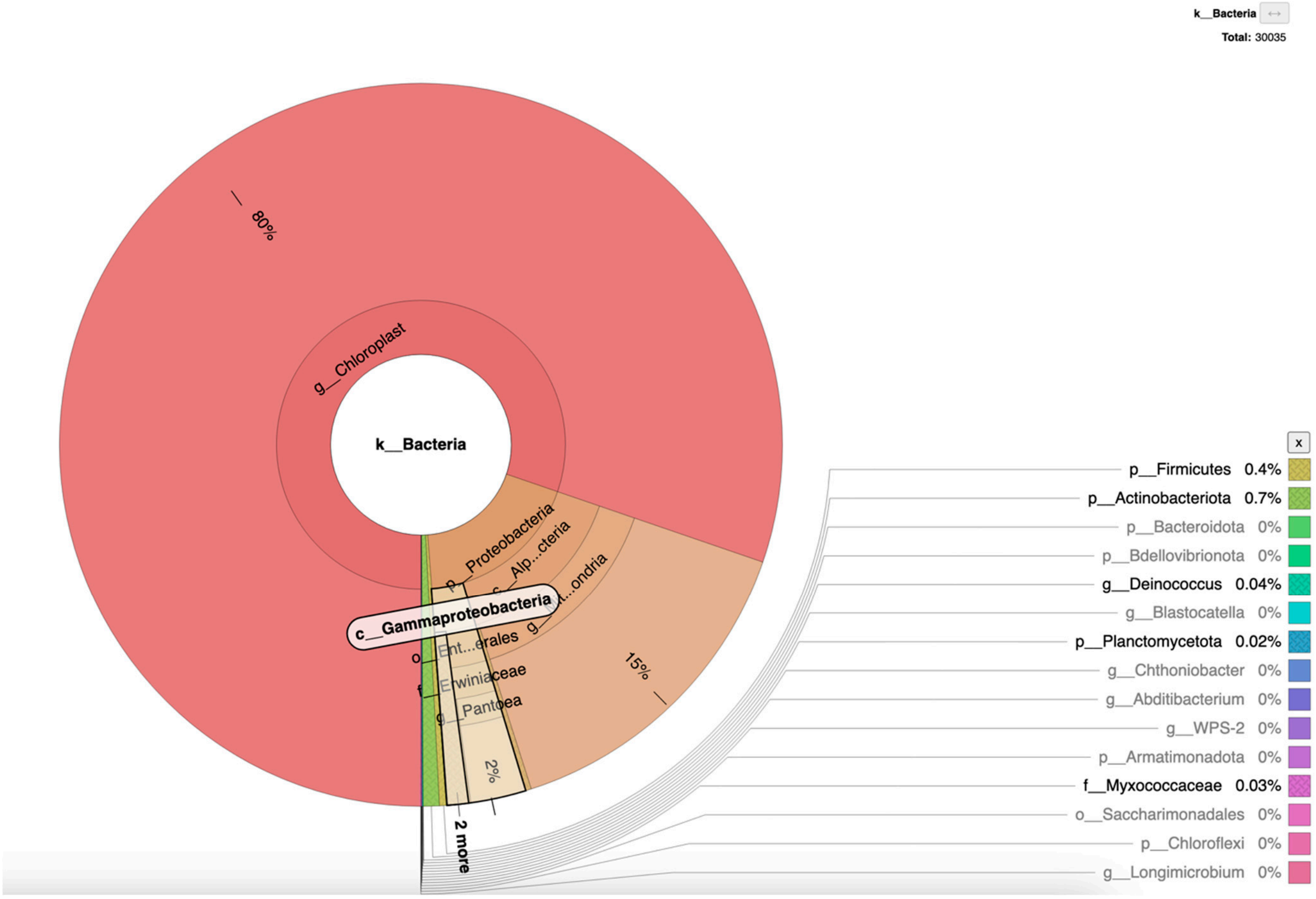
k__Bacteria
Total: 30035
80%
g__Chloroplast
k__Bacteria
p__Proteobacteria
Alp...cteria
...ondria
c__Gammaproteobacteria
Ent...erales
Erwiniaceae
Pantoea
15%
2%
2 more
p__Firmicutes 0.4%
p__Actinobacteriota 0.7%
p__Bacteroidota 0%
p__Bdellovibrionota 0%
g__Deinococcus 0.04%
g__Blastocatella 0%
p__Planctomycetota 0.02%
g__Chthoniobacter 0%
g__Abditibacterium 0%
g__WPS-2 0%
p__Armatimonadota 0%
f__Myxococcaceae 0.03%
o__Saccharimonadales 0%
p__Chloroflexi 0%
g__Longimicrobium 0%

B-1

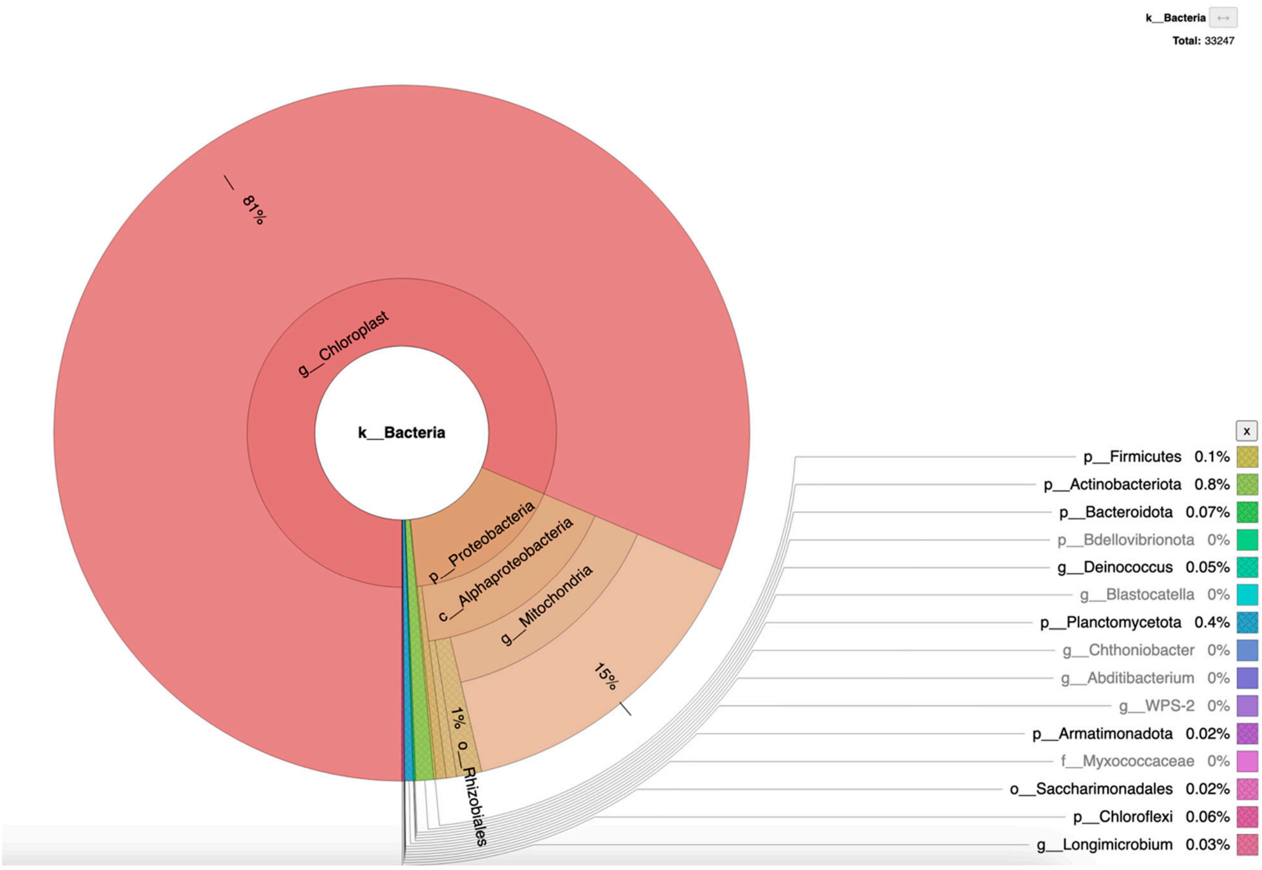

k__Bacteria
Total: 33247
81%
g__Chloroplast
k__Bacteria
p__Proteobacteria
c__Alphaproteobacteria
g__Mitochondria
15%
1%
o__Rhizobiales
p__Firmicutes 0.1%
p__Actinobacteriota 0.8%
p__Bacteroidota 0.07%
p__Bdellovibrionota 0%
g__Deinococcus 0.05%
g__Blastocatella 0%
p__Planctomycetota 0.4%
g__Chthoniobacter 0%
g__Abditibacterium 0%
g__WPS-2 0%
p__Armatimonadota 0.02%
f__Myxococcaceae 0%
o__Saccharimonadales 0.02%
p__Chloroflexi 0.06%
g__Longimicrobium 0.03%

B-2
