# Supplementary material for: Status of Cassava Witches’ Broom Disease in the Philippines and Identification of Potential Pathogens by Metagenomic Analysis
Source: Biology (Basel). 2024 Jul 15;13(7):522. doi: 10.3390/biology13070522 (PMC11273669; doi:10.3390/biology13070522)
Supplement: Supplementary file 1 [file biology-13-00522-s001.zip › Figure S5-Microbial taxonomic classification of shotgun metagenomics reads using Kaiju.pdf]

Figure S5. Microbial taxonomic classification of shotgun metagenomic reads using Kaiju

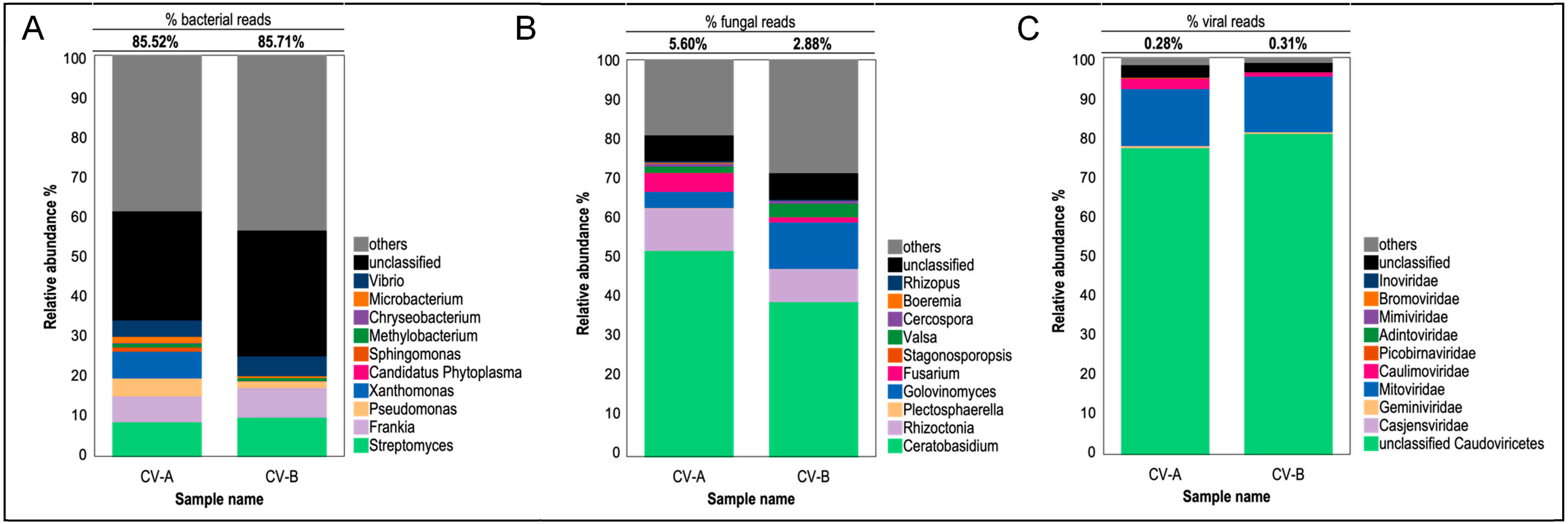

Relative abundance graph of **(A)** fungi**, (B)** bacteria, and **(C)** viral genomic reads in bitter gourd, sponge gourd, and cassava. Detected bacterial (genus), fungal (genus) and viral classifications (family) are color-differentiated. The percentage of fungal and viral reads against total reads is indicated above the bar chart. CV-A, genomic reads from CWBD-affected cassava from Bukidnon; CV-B, genomic reads from CWBD-affected cassava from Isabela.
