# Supplementary material for: Status of Cassava Witches’ Broom Disease in the Philippines and Identification of Potential Pathogens by Metagenomic Analysis
Source: Biology (Basel). 2024 Jul 15;13(7):522. doi: 10.3390/biology13070522 (PMC11273669; doi:10.3390/biology13070522)
Supplement: Supplementary file 1 [file biology-13-00522-s001.zip › Figure S6-Krona Pie charts of Kaiju classification of genomic reads, contrigs, and transcripts.pdf]

Figure S6. Krona Pie charts of Kaiju classification of genomic reads, contigs, and transcripts

I. Genomic reads and contigs

A. Fungi

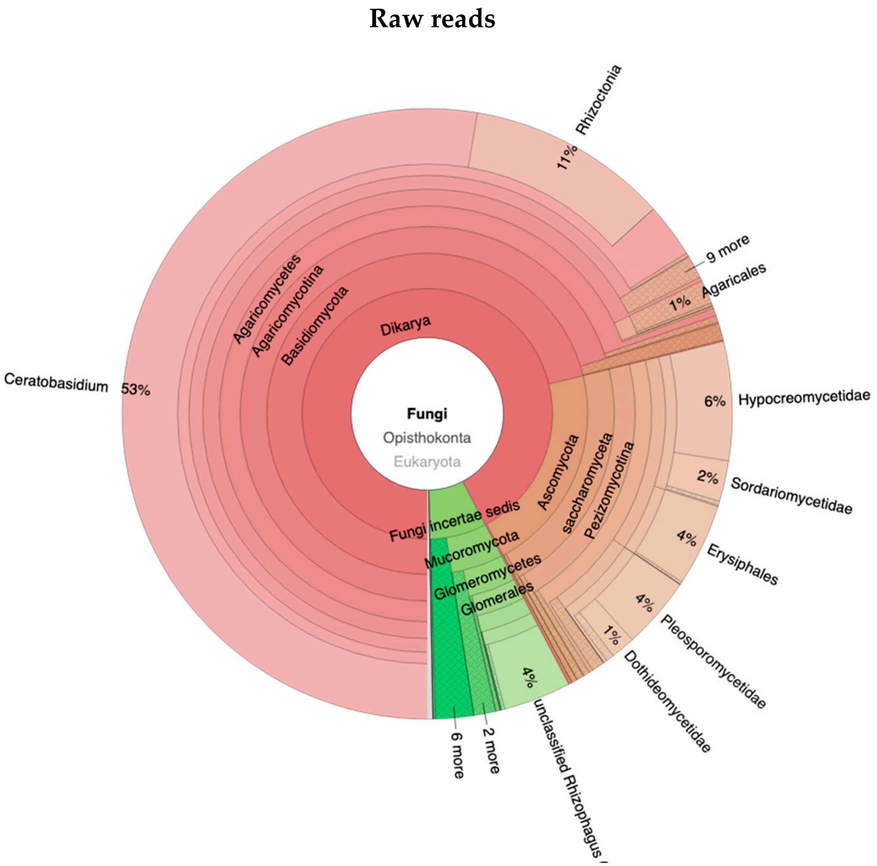

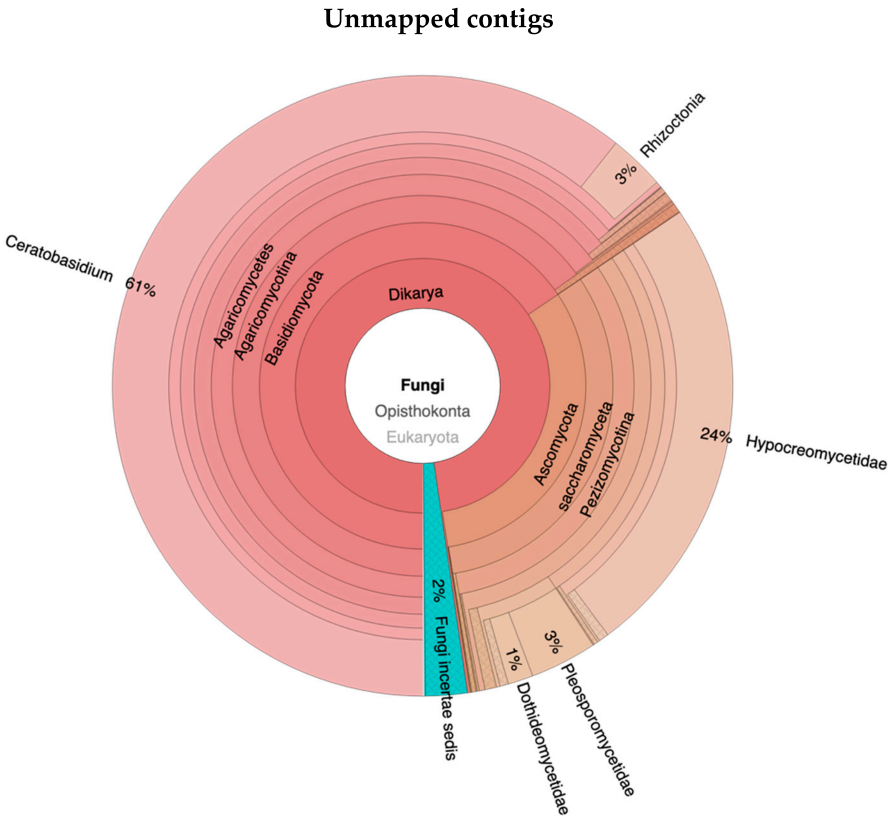

CV-A

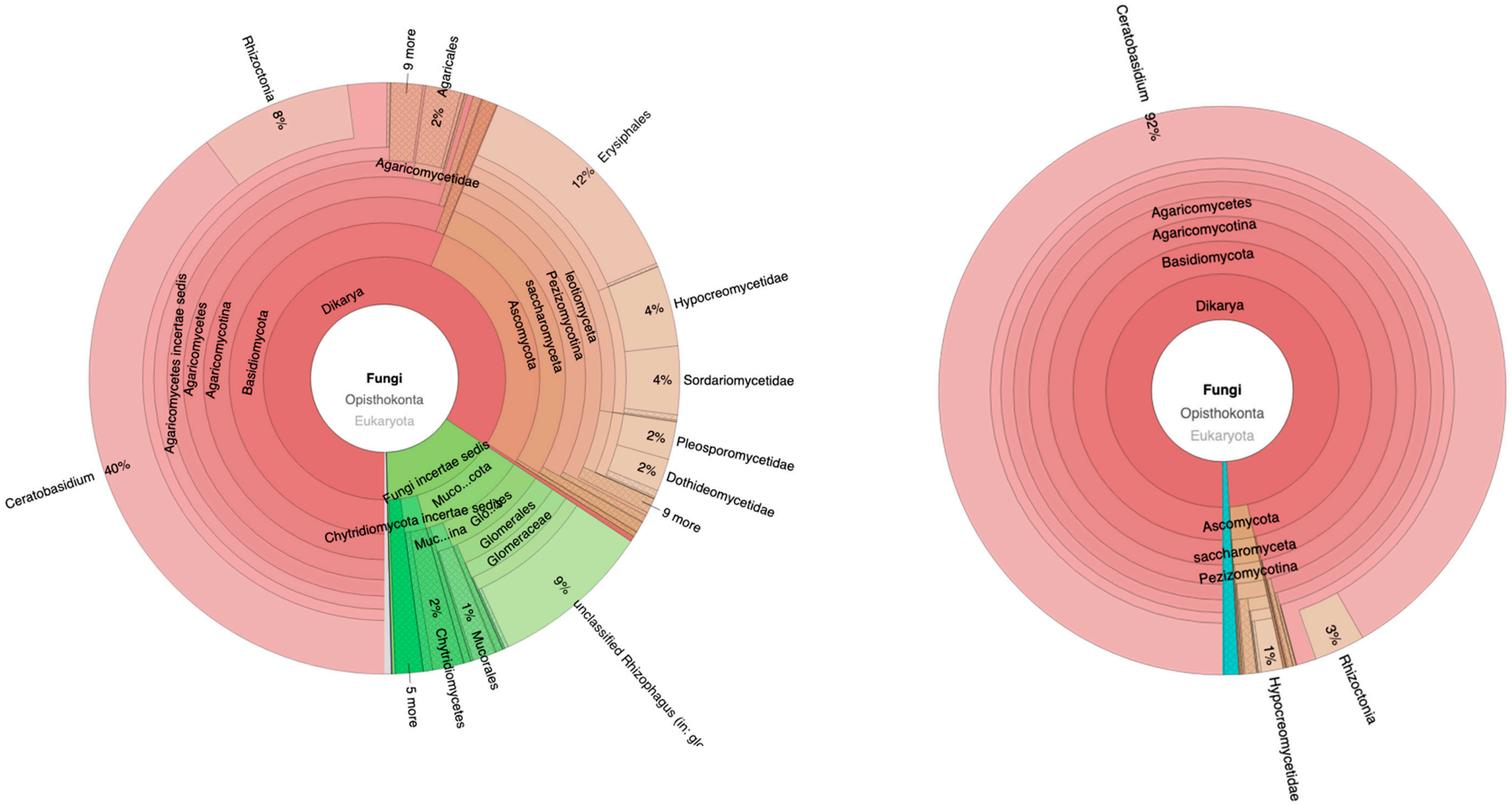
Fungi
Opisthokonta
Eukaryota
Dikarya
Basidiomycota
Agaricomycotina
Agaricomycetes
Agaricomycetes incertae sedis
Ceratobasidium 40%
Rhizoctonia 8%
9 more
Agaricales 2%
Agaricomycetidae
Erysiphales 12%
Ascomycota
saccharomyceta
Pezizomycotina
leotiomyceta
Hypocreomycetidae 4%
Sordariomycetidae 4%
Pleosporomycetidae 2%
Dothideomycetidae 2%
9 more
Fungi incertae sedis
Muco...cota
Chytridiomycota incertae sedis
Muc...ina
Glomerales
Glomeraceae
9% unclassified Rhizophagus (in: gl
2% Chytridiomycetes
1% Mucorales
5 more
Ceratobasidium 92%
Agaricomycetes
Agaricomycotina
Basidiomycota
Dikarya
Fungi
Opisthokonta
Eukaryota
Ascomycota
saccharomyceta
Pezizomycotina
1% Hypocreomycetidae
3% Rhizoctonia

CV-B

B. Bacteria

Raw reads

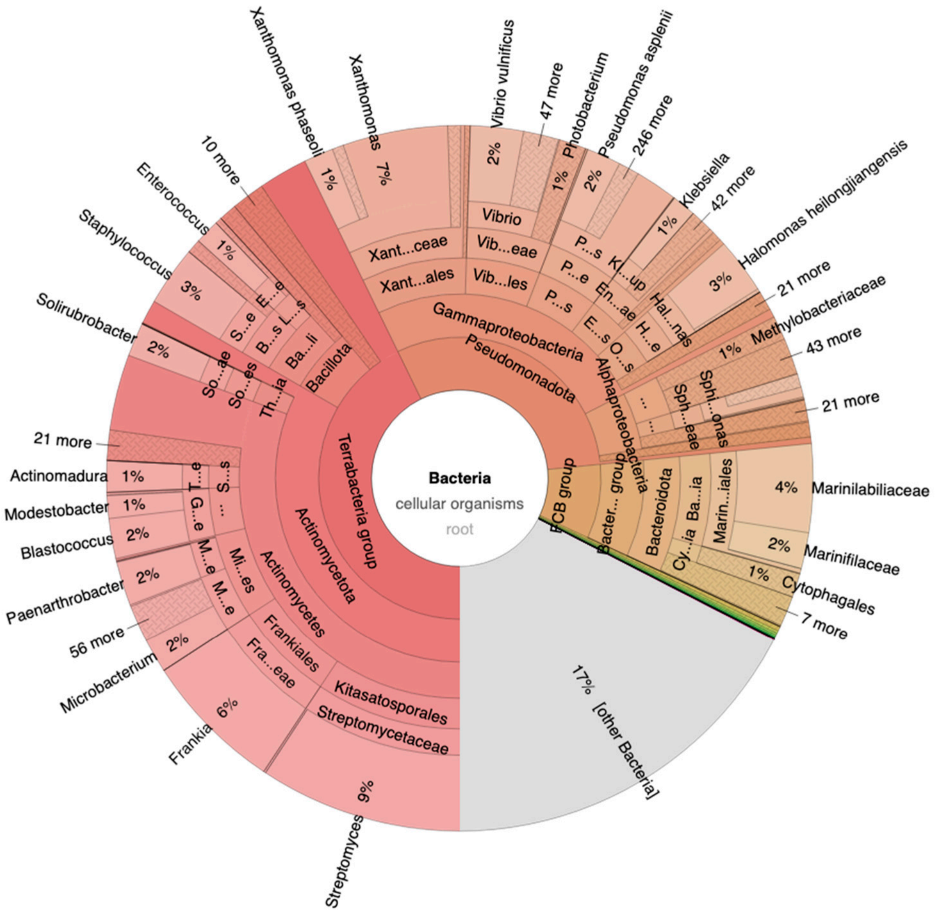

Unmapped contigs

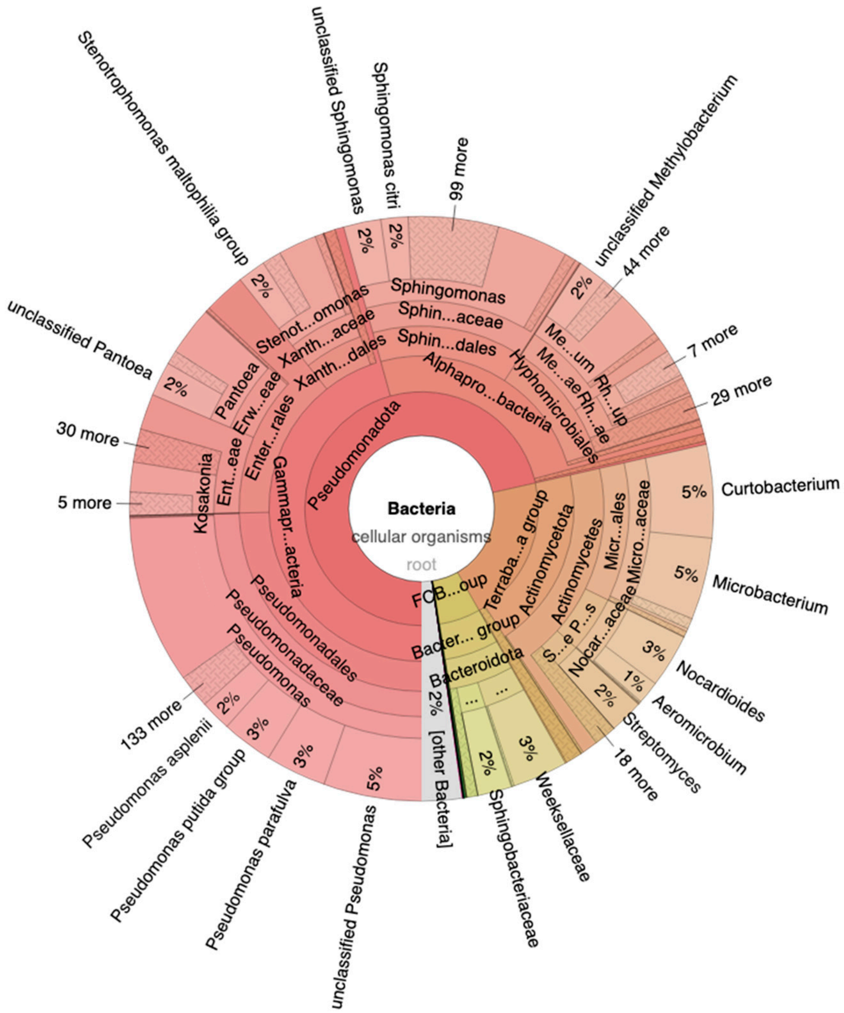

CV-A

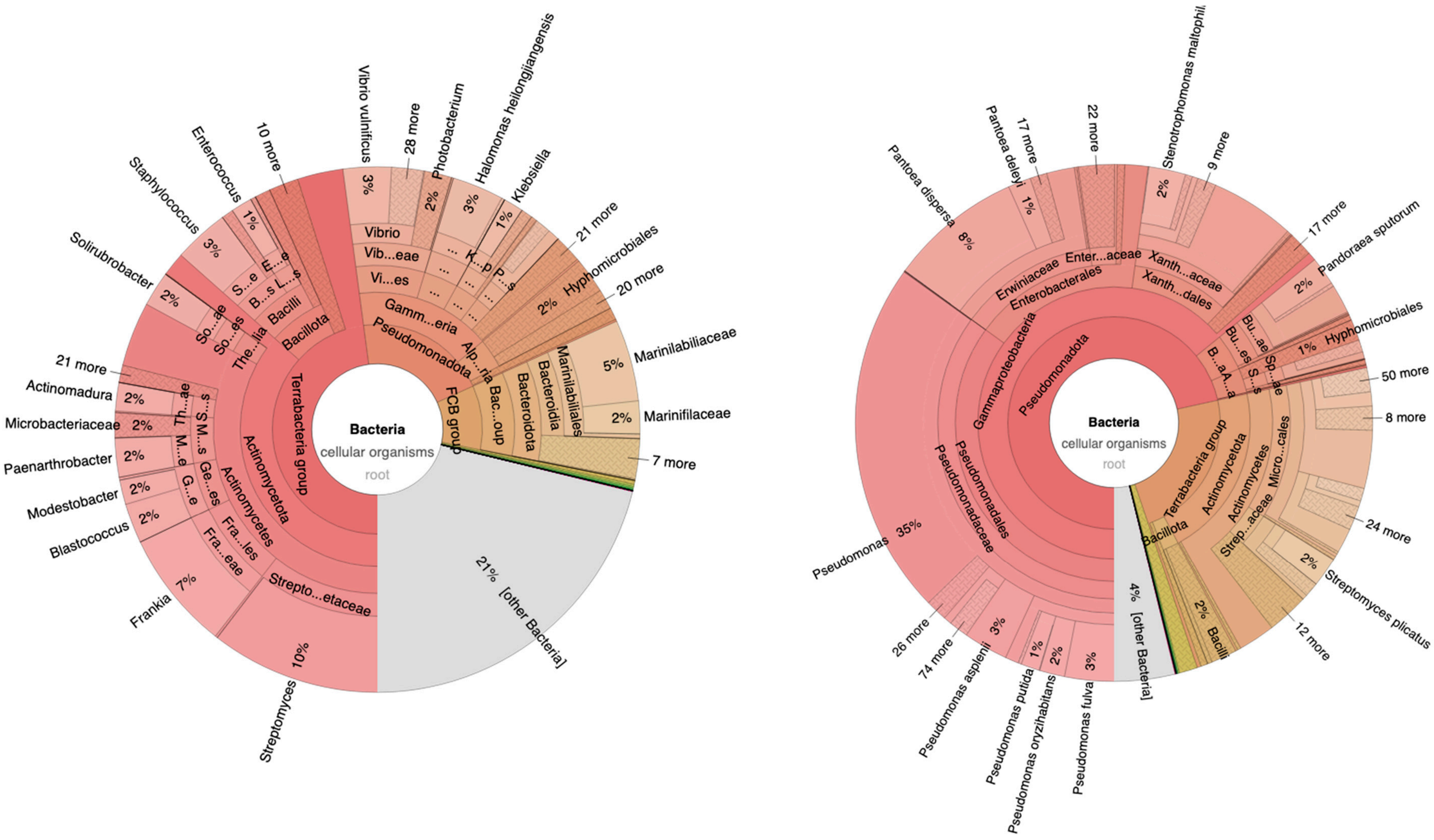
Bacteria
cellular organisms
root
Terrabacteria group
Actinomycetota
Actinomycetes
Pseudomonadota
Gamm...eria
Alp...ria
FCB group
Bac...oup
Bacteroidota
Bacteroidia
Marinilabiliales
Marinilabiliaceae
Marinifilaceae
7 more
21% [other Bacteria]
Streptomyces
10%
Strepto...etaceae
Frankia
7%
Fra...eae
Fra...les
Blastococcus
Modestobacter
Paenarthrobacter
Microbacteriaceae
Actinomadura
21 more
Solirubrobacter
Staphylococcus
Enterococcus
10 more
Bacillota
Bacilli
Vibrio vulnificus
28 more
Photobacterium
Halomonas heilongjiangensis
Klebsiella
21 more
Hyphomicrobiales
20 more
Vibrio
Vib...eae
Vi...es
5%
3%
2%
1%
Bacteria
cellular organisms
root
Pseudomonadota
Gammaproteobacteria
Pseudomonadales
Pseudomonadaceae
Pseudomonas
35%
Enterobacterales
Erwiniaceae
Enter...aceae
Xanth...aceae
Xanth...dales
Pantoea dispersa
8%
Pantoea deleyi
17 more
22 more
Stenotrophomonas maltophili
9 more
17 more
Pandoraea sputorum
Hyphomicrobiales
50 more
8 more
24 more
Streptomyces plicatus
12 more
Terrabacteria group
Actinomycetota
Actinomycetes
Micro...cales
Strep...aceae
Bacillota
Bacilli
4% [other Bacteria]
Pseudomonas fulva
Pseudomonas oryzihabitans
Pseudomonas putida
Pseudomonas asplenii
74 more
26 more
3%
2%
1%

CV-B

c. Virus

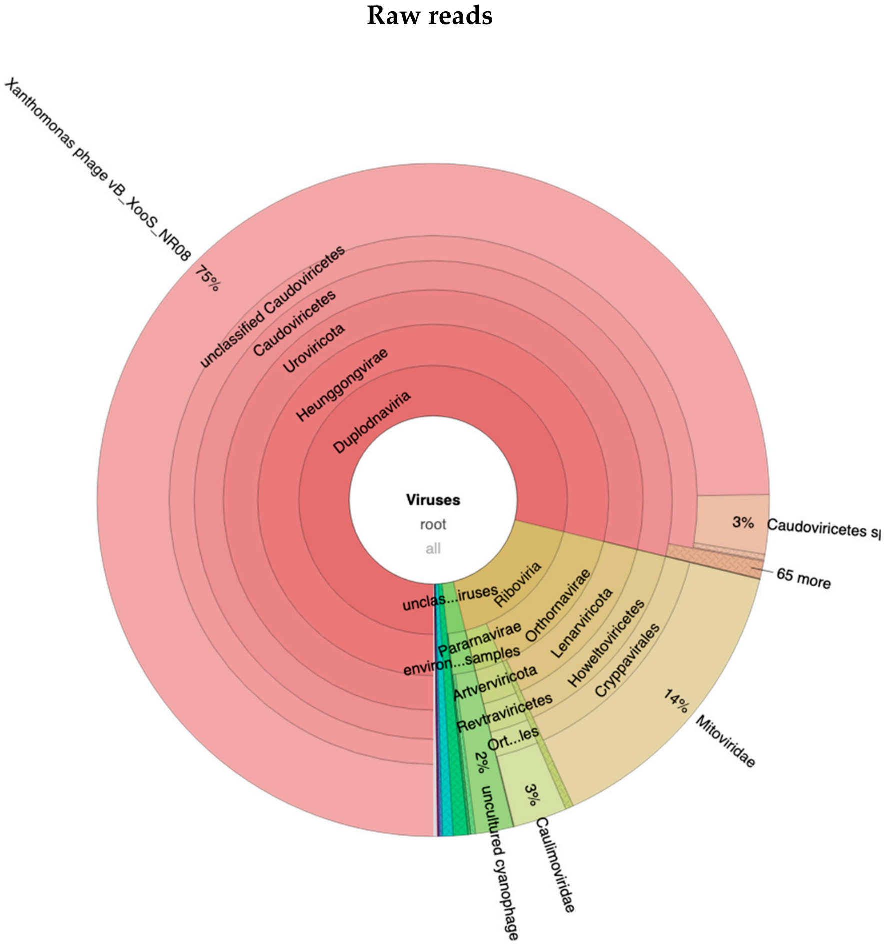

Raw reads
Xanthomonas phage vB_XooS_NR08
75%
unclassified Caudoviricetes
Caudoviricetes
Uroviricota
Heunggongvirae
Duplodnaviria
Viruses
root
all
3%
Caudoviricetes s
65 more
unclas...iruses
Riboviria
Orthornavirae
Lenarviricota
Howeltoviricetes
Cryppavirales
Pararnavirae
environ...samples
Artverviricota
Revtraviricetes
Ort...les
14%
Mitoviridae
2%
uncultured cyanophage
3%
Caulimoviridae

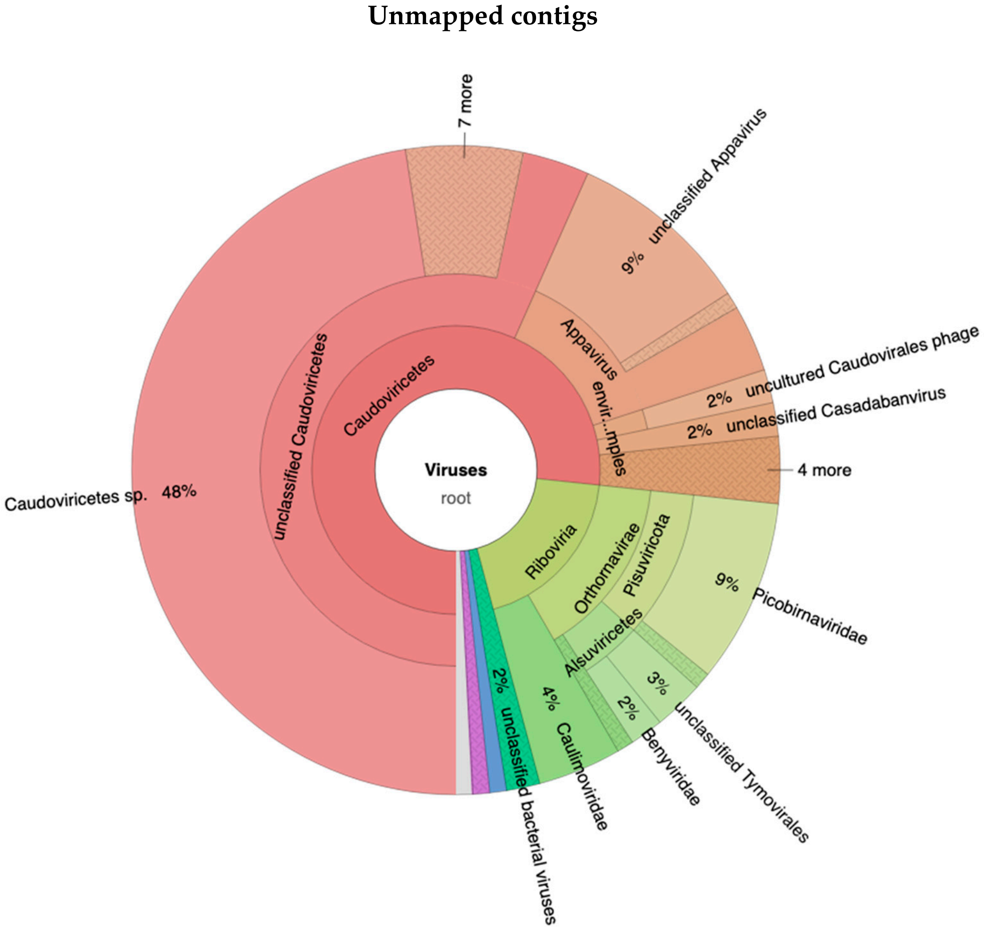

Unmapped contigs
7 more
unclassified Appavirus
9%
Appavirus
envir...mples
2%
uncultured Caudovirales phage
2%
unclassified Casadabanvirus
4 more
Caudoviricetes sp.
48%
unclassified Caudoviricetes
Caudoviricetes
Viruses
root
Riboviria
Orthornavirae
Pisuviricota
9%
Picobirnaviridae
Alsuviricetes
3%
2%
unclassified Tymovirales
Benyviridae
4%
Caulimoviridae
2%
unclassified bacterial viruses

CV-A

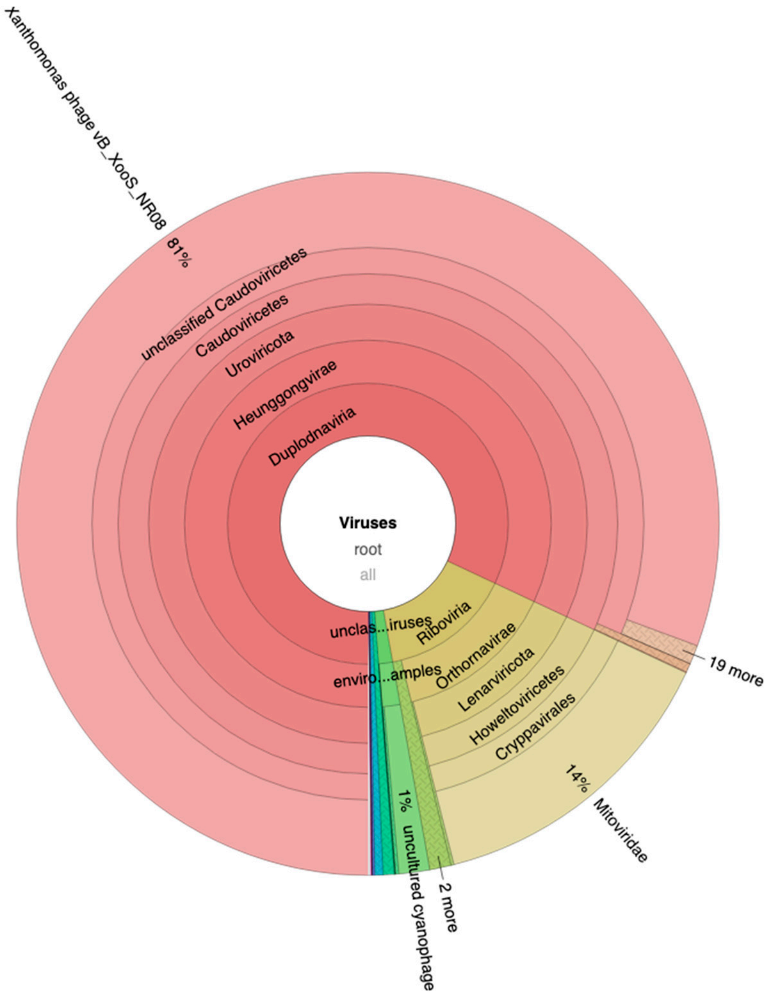
Xanthomonas phage vB_XooS_NR08
81%
unclassified Caudoviricetes
Caudoviricetes
Uroviricota
Heunggongvirae
Duplodnaviria
Viruses
root
all
unclas...iruses
Riboviria
enviro...amples
Orthornavirae
Lenarviricota
Howeltoviricetes
Cryppavirales
14%
Mitoviridae
19 more
1%
uncultured cyanophage
2 more

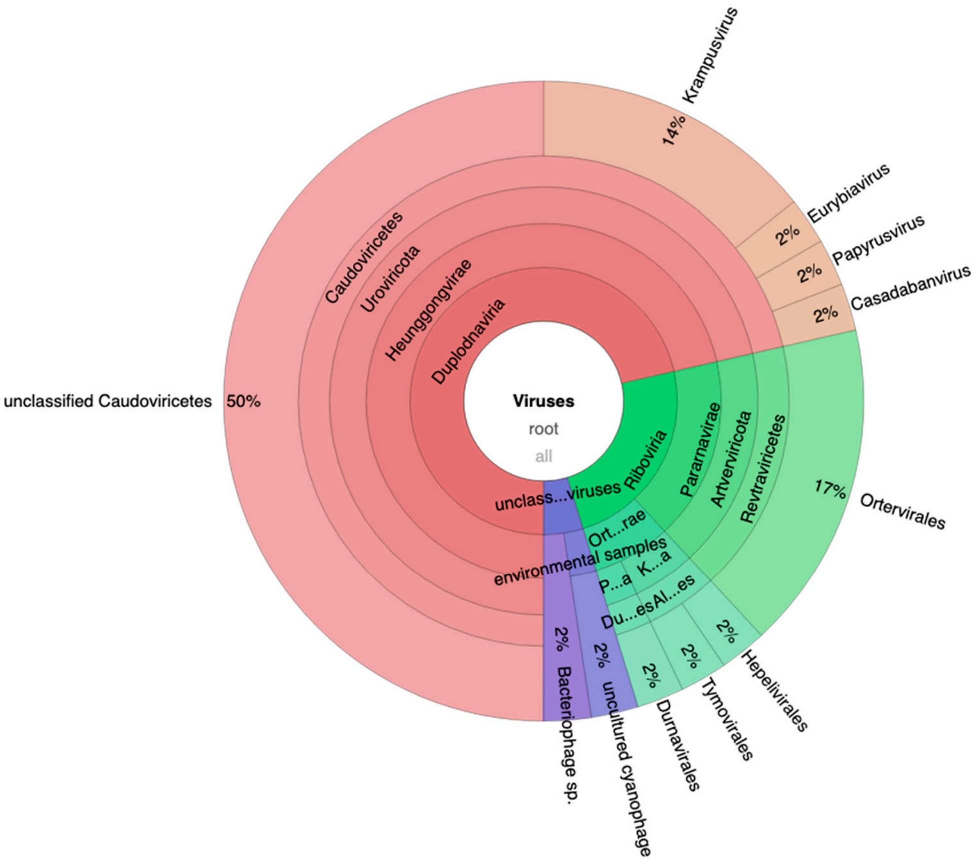
Krampusvirus
14%
Eurybiavirus
2%
Papyrusvirus
2%
Casadabanvirus
2%
Caudoviricetes
Uroviricota
Heunggongvirae
Duplodnaviria
Viruses
root
all
unclassified Caudoviricetes 50%
unclass...viruses
Riboviria
Pararnavirae
Artverviricota
Revtraviricetes
17%
Ortervirales
Ort...rae
environmental samples
P...a
K...a
Du...es
Al...es
2%
Bacteriophage sp.
2%
uncultured cyanophage
2%
Durnavirales
2%
Tymovirales
2%
Hepelivirales

CV-B

II. Transcripts

A. Fungi

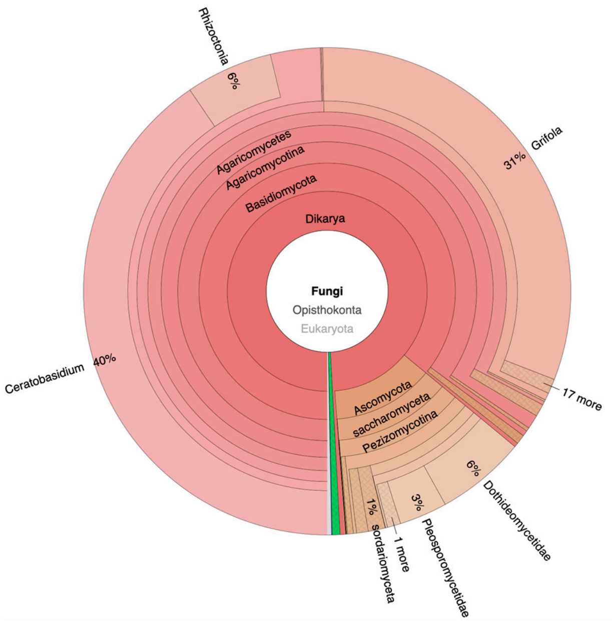

I-1

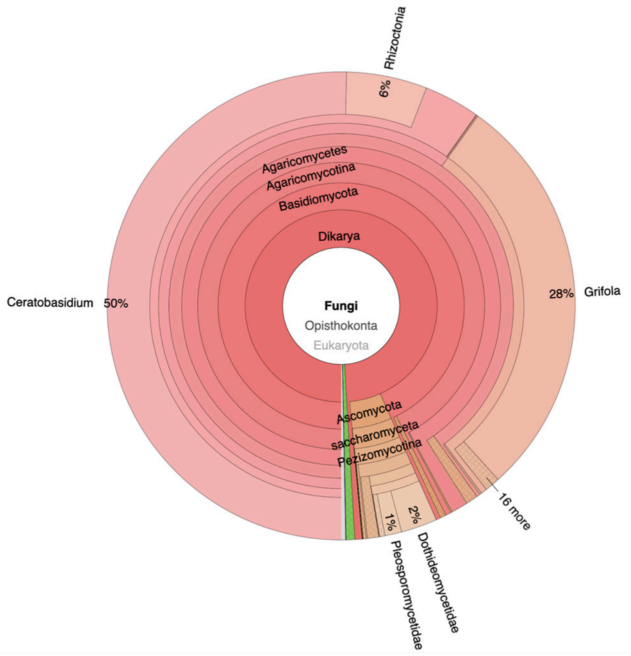

I-2

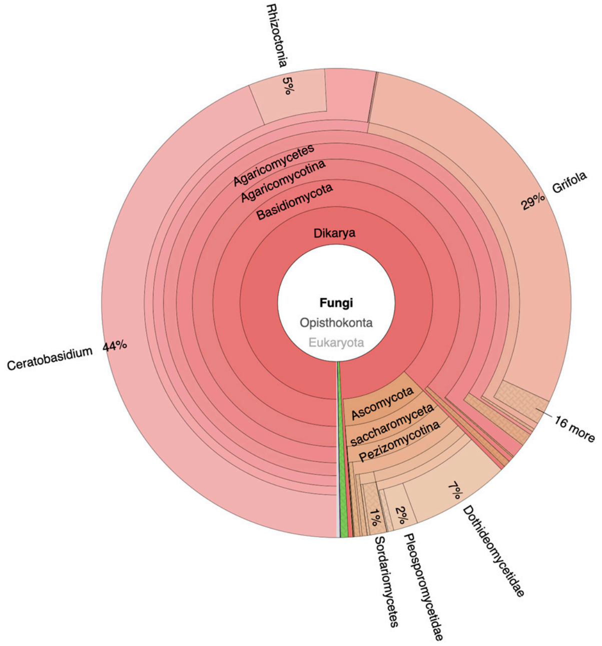

I-3

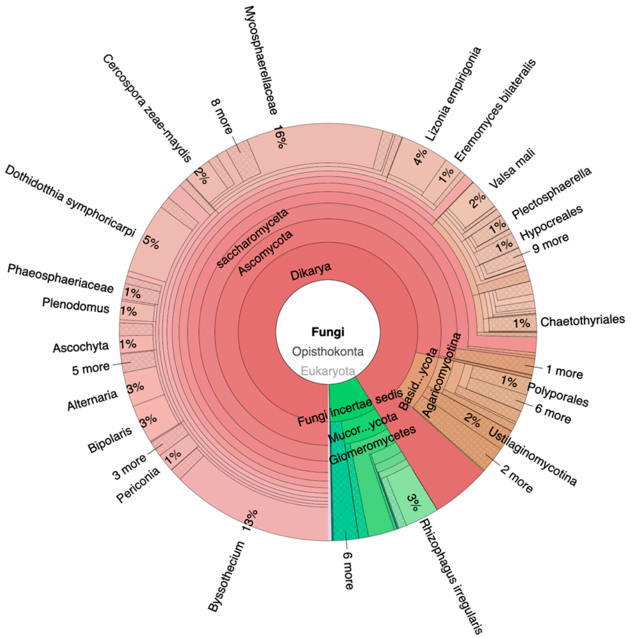
Fungi
Opisthokonta
Eukaryota
Dikarya
Ascomycota
saccharomyceta
Mycosphaerellaceae
16%
8 more
Cercospora zeae-maydis
2%
Dothidotthia symphoricarpi
5%
Phaeosphaeriaceae
1%
Plenodomus
1%
Ascochyta
1%
5 more
Alternaria
3%
Bipolaris
3%
3 more
Periconia
1%
Byssothecium
13%
6 more
Lizonia empirigonia
4%
Eremomyces bilateralis
1%
Valsa mali
2%
Plectosphaerella
1%
Hypocreales
1%
9 more
Chaetothyriales
1%
1 more
Polyporales
1%
6 more
2%
Ustilaginomycotina
2 more
Basid...ycota
Agaricomycotina
Fungi incertae sedis
Mucor...ycota
Glomeromycetes
3%
Rhizophagus irregularis

H-1

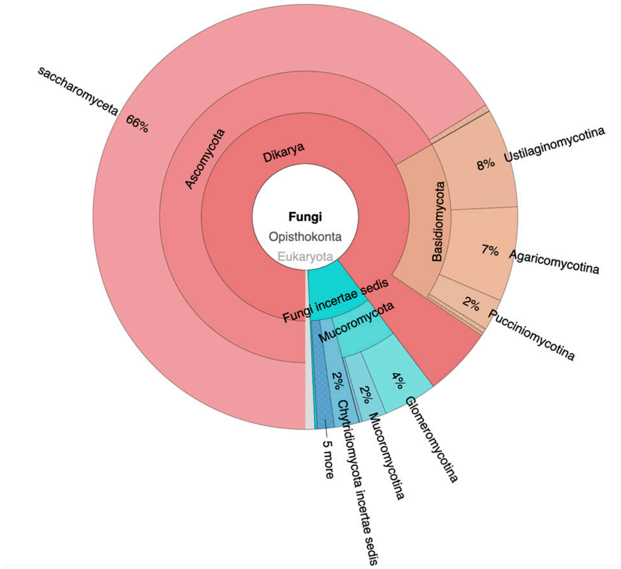
Fungi
Opisthokonta
Eukaryota
Dikarya
Ascomycota
saccharomyceta
66%
Basidiomycota
8%
Ustilaginomycotina
7%
Agaricomycotina
2%
Pucciniomycotina
Fungi incertae sedis
Mucoromycota
4%
Glomeromycotina
2%
Mucoromycotina
2%
Chytridiomycota incertae sedis
5 more

H-2

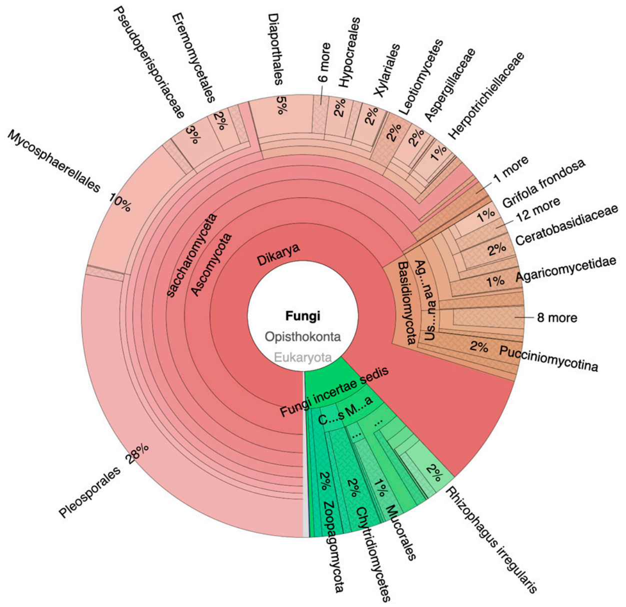
Fungi
Opisthokonta
Eukaryota
Dikarya
Ascomycota
saccharomyceta
Pseudoperisporiaceae
3%
Eremomycetales
2%
Diaporthales
5%
6 more
Hypocreales
2%
Xylariales
2%
Leotiomycetes
2%
Aspergillaceae
2%
Herpotrichiellaceae
1%
Mycosphaerellales
10%
Pleosporales
28%
1 more
Grifola frondosa
1%
12 more
Ceratobasidiaceae
2%
Agaricomycetidae
1%
8 more
2%
Pucciniomycotina
Basidiomycota
Ag...na
Us...na
Fungi incertae sedis
C...s M...a
2%
Zoopagomycota
2%
Chytridiomycetes
1%
Mucorales
2%
Rhizophagus irregularis

H-3

B. Bacteria

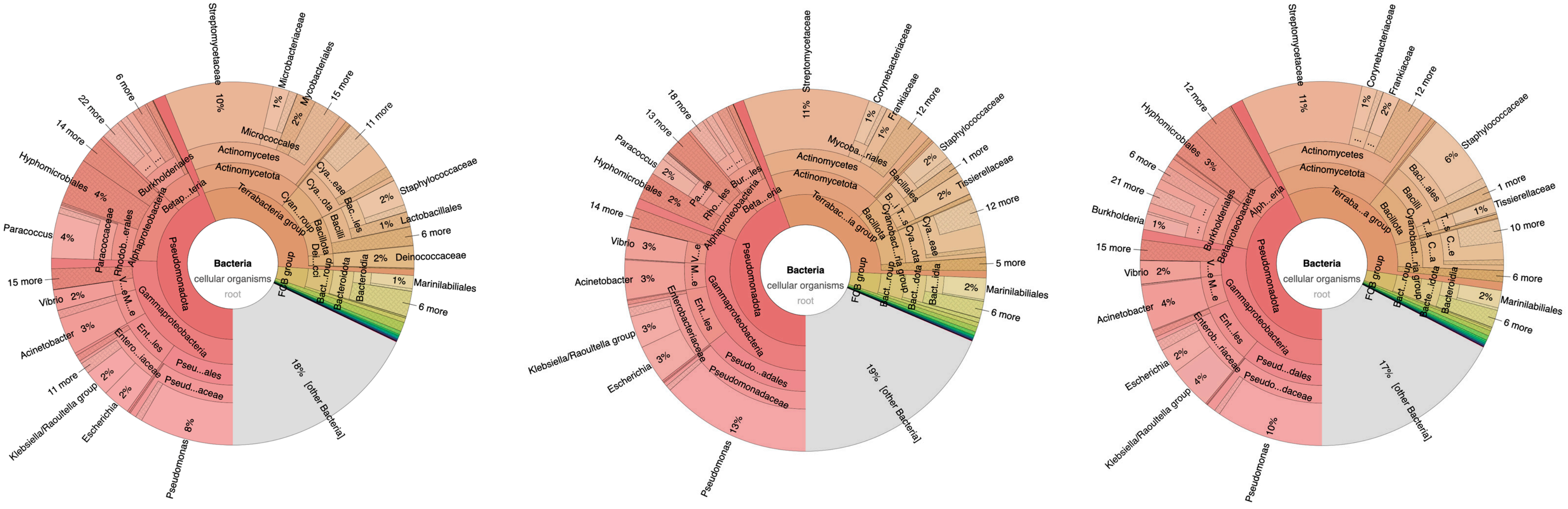

I-1 I-2 I-3

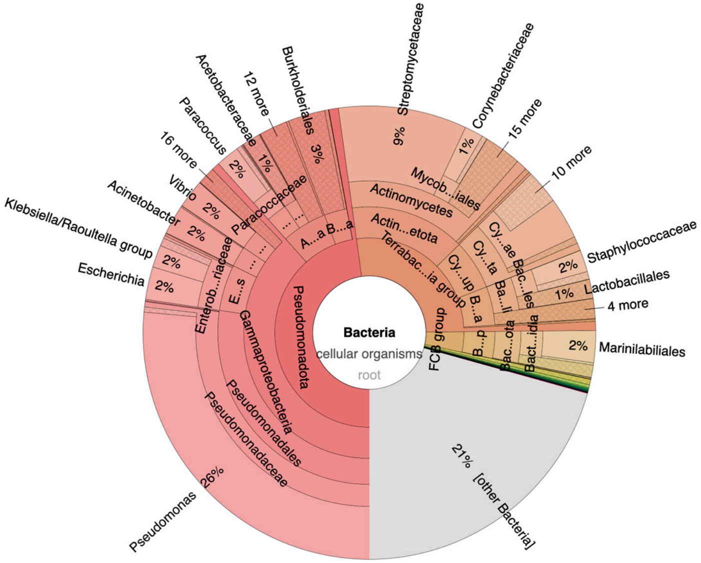
Bacteria
cellular organisms
root
Pseudomonas
26%
21% [other Bacteria]
Escherichia
Klebsiella/Raoultella group
Acinetobacter
Vibrio
16 more
Paracoccus
Acetobacteraceae
12 more
Burkholderiales
Streptomycetaceae
Corynebacteriaceae
15 more
10 more
Staphylococcaceae
Lactobacillales
4 more
Marinilabiliales
Pseudomonadota
Gammaproteobacteria
Pseudomonadales
Pseudomonadaceae
Actinomycetes
FCB group

H-1

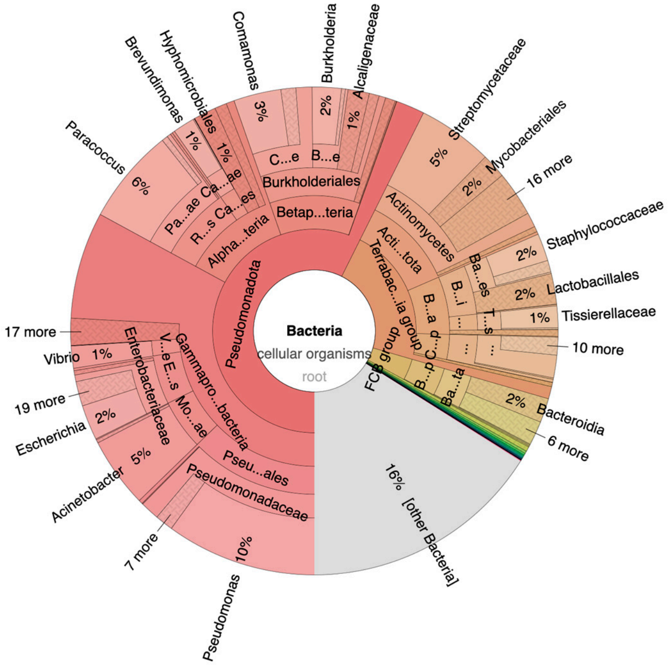
Bacteria
cellular organisms
root
Pseudomonas
10%
16% [other Bacteria]
7 more
Acinetobacter
Escherichia
19 more
Vibrio
17 more
Paracoccus
Brevundimonas
Hyphomicrobiales
Comamonas
Burkholderia
Alcaligenaceae
Streptomycetaceae
Mycobacteriales
16 more
Staphylococcaceae
Lactobacillales
Tissierellaceae
10 more
Bacteroidia
6 more
Pseudomonadota
Burkholderiales
Enterobacteriaceae
Pseudomonadaceae
FCB group

H-2

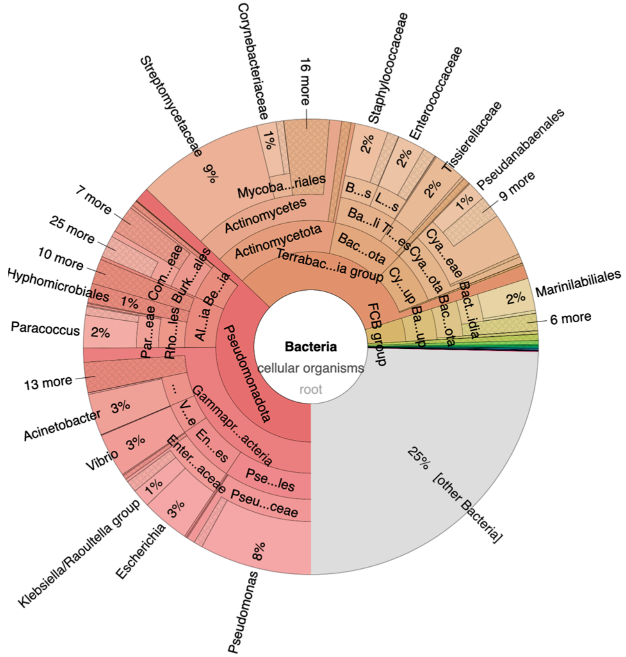
Bacteria
cellular organisms
root
Pseudomonas
8%
25% [other Bacteria]
Escherichia
Klebsiella/Raoultella group
Vibrio
Acinetobacter
13 more
Paracoccus
Hyphomicrobiales
10 more
25 more
7 more
Streptomycetaceae
Corynebacteriaceae
16 more
Staphylococcaceae
Enterococcaceae
Tissierellaceae
Pseudanabaenales
9 more
Marinilabiliales
6 more
Pseudomonadota
Actinomycetota
FCB group

H-3

C. Virus

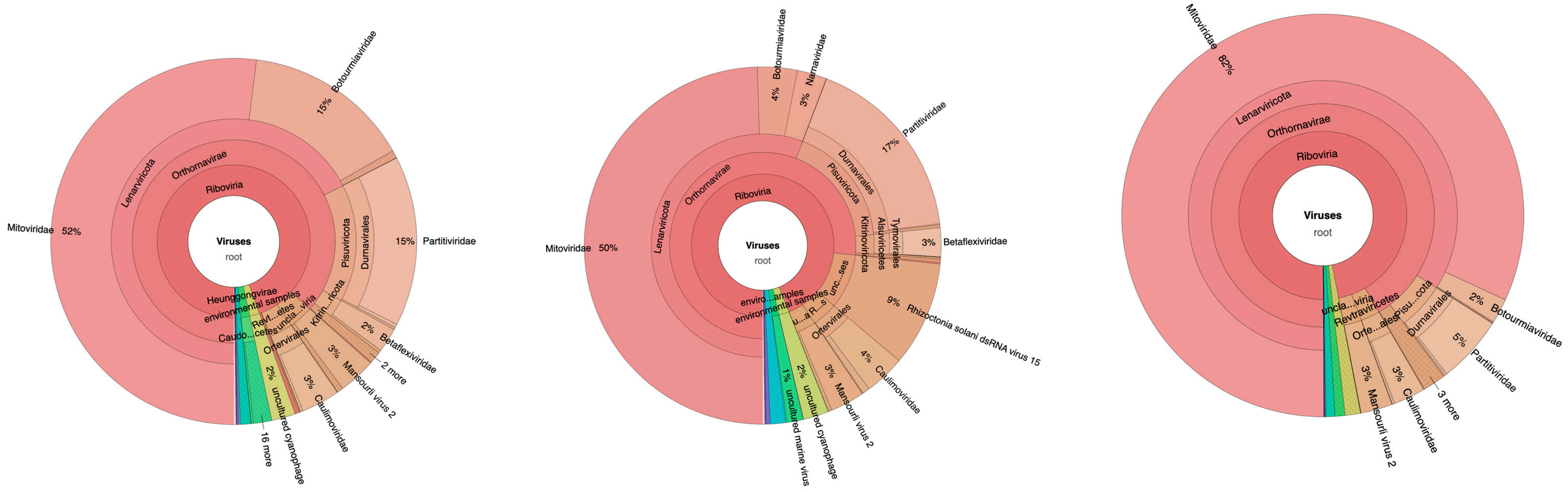

I-1 I-2 I-3

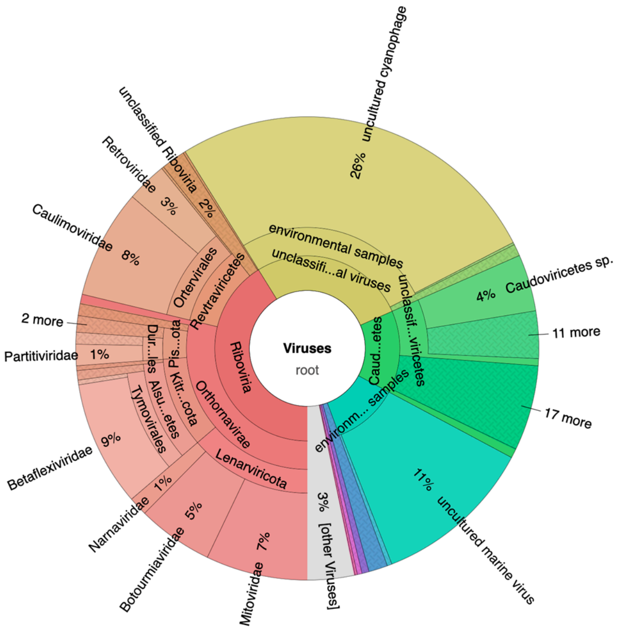
Viruses
root
26% uncultured cyanophage
environmental samples
unclassifi...al viruses
unclassified Riboviria 2%
Retroviridae 3%
Caulimoviridae 8%
Ortervirales
Revtraviricetes
Riboviria
2 more
Partitiviridae 1%
Dur...les
Pis...ota
Alsu...etes
Kitr...cota
Tymovirales
Orthornavirae
Betaflexiviridae 9%
Narnaviridae 1%
Lenarviricota
Botourmiaviridae 5%
Mitoviridae 7%
3% [other Viruses]
environm... samples
Caud...etes
unclassif...viricetes
4% Caudoviricetes sp.
11 more
17 more
11% uncultured marine virus

H-1

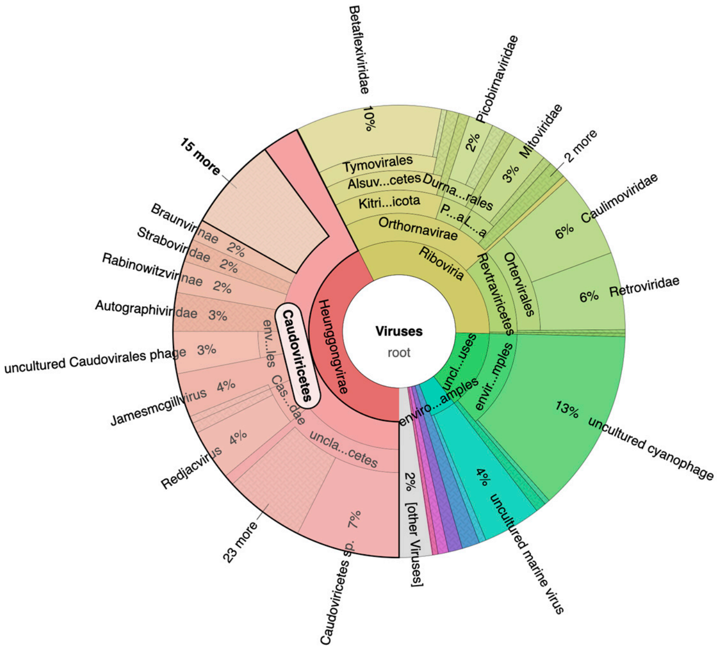
Viruses
root
Betaflexiviridae 10%
Picobirnaviridae 2%
Mitoviridae 3%
2 more
Caulimoviridae 6%
Retroviridae 6%
Tymovirales
Alsuv...cetes
Durna...rales
Kitri...icota
P...aL...a
Orthornavirae
Riboviria
Revtraviricetes
Ortervirales
15 more
Braunvirinae 2%
Strabovirdae 2%
Rabinowitzvirinae 2%
Autographivirdae 3%
uncultured Caudovirales phage 3%
Jamesmcgillvirus 4%
Redjacvirus 4%
23 more
Caudoviricetes sp. 7%
Caudoviricetes
Heunggongvirae
env...les
Cas...dae
uncla...cetes
2% [other Viruses]
enviro...amples
uncl...uses
envir...mples
13% uncultured cyanophage
4% uncultured marine virus

H-2

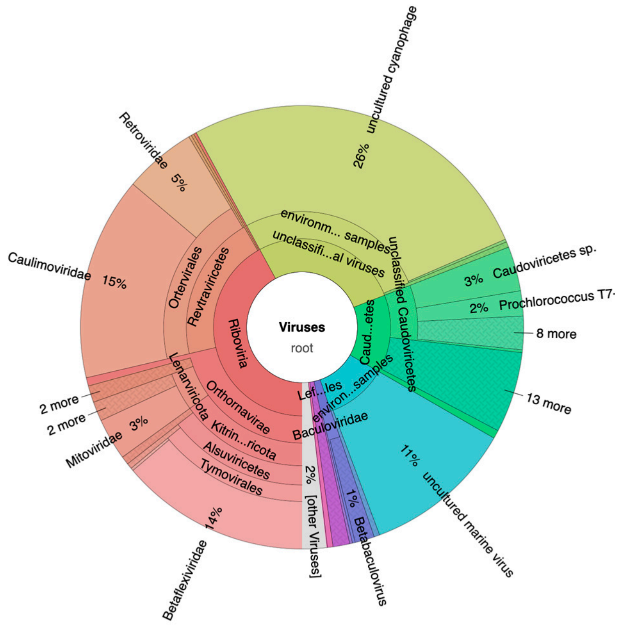
Viruses
root
26% uncultured cyanophage
environm... samples
unclassifi...al viruses
unclassified Caudoviricetes
Retroviridae 5%
Caulimoviridae 15%
Ortervirales
Revtraviricetes
Riboviria
3% Caudoviricetes sp.
2% Prochlorococcus T7-
8 more
13 more
Caud...etes
environ...samples
Lef...les
Baculoviridae
Orthornavirae
Kitrin...ricota
Alsuviricetes
Tymovirales
Lenarviricota
2 more
2 more
Mitoviridae 3%
Betaflexiviridae 14%
2% [other Viruses]
1% Betabaculovirus
11% uncultured marine virus

H-3
