# Supplementary material for: Status of Cassava Witches’ Broom Disease in the Philippines and Identification of Potential Pathogens by Metagenomic Analysis
Source: Biology (Basel). 2024 Jul 15;13(7):522. doi: 10.3390/biology13070522 (PMC11273669; doi:10.3390/biology13070522)
Supplement: Supplementary file 1 [file biology-13-00522-s001.zip › Raw gel Images.pdf]

Supplementary information. Raw gel images

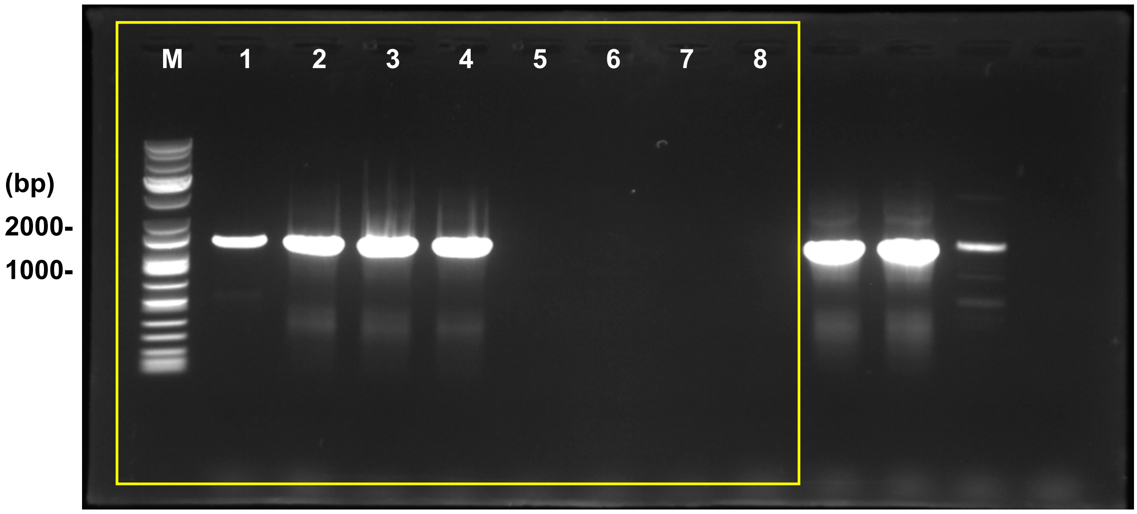

Figure 4A – Cassava (CV)

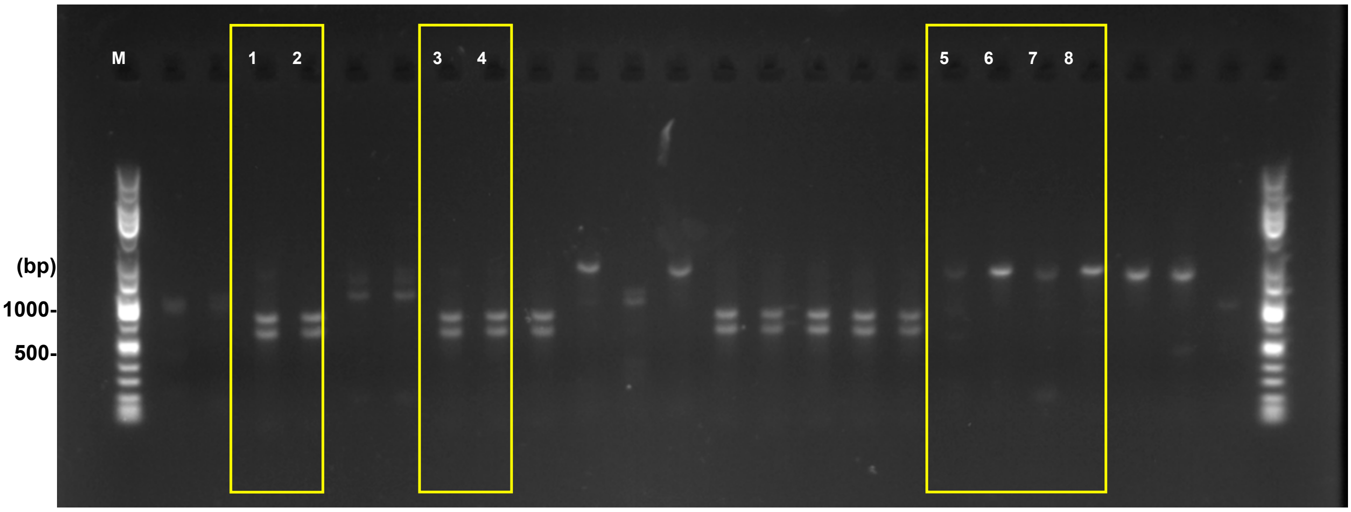

Figure 4B – Cassava (CV)

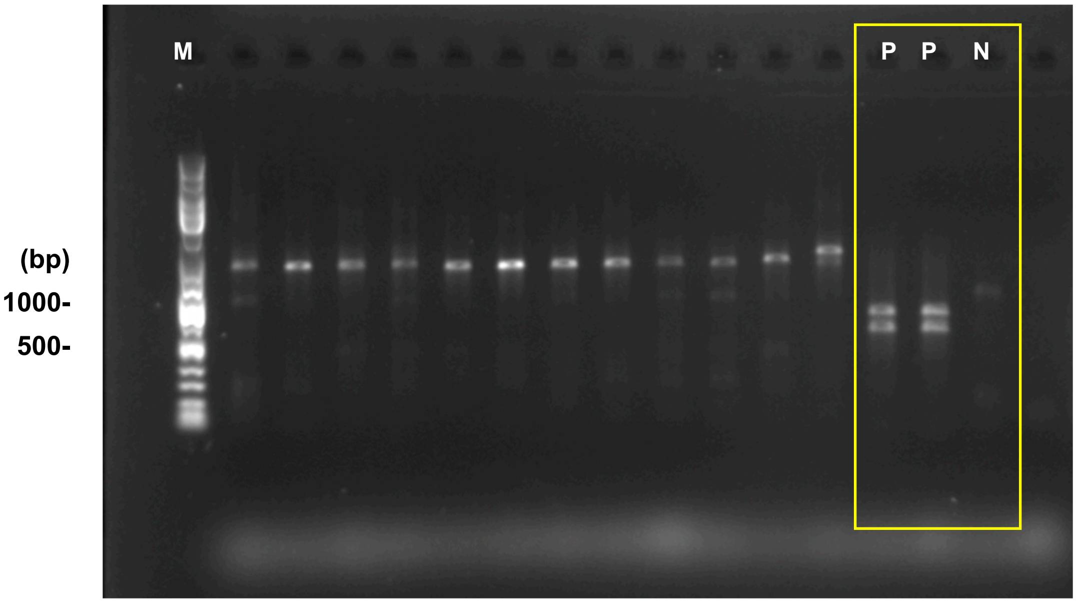

Figure 4B – Controls (*Ca*. P. luffae, *Ca*. P. mali and no template control)

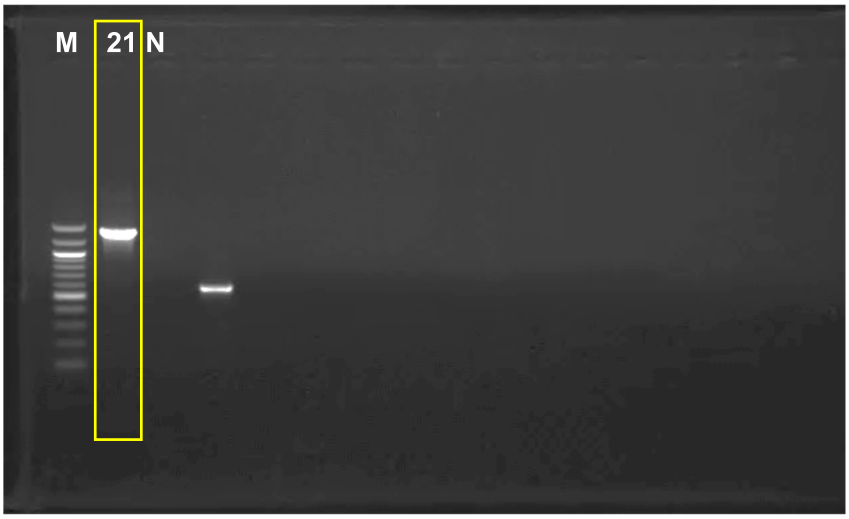

Figure 4B – Controls (asymptomatic *Ca*. P. pruni positive cassava)

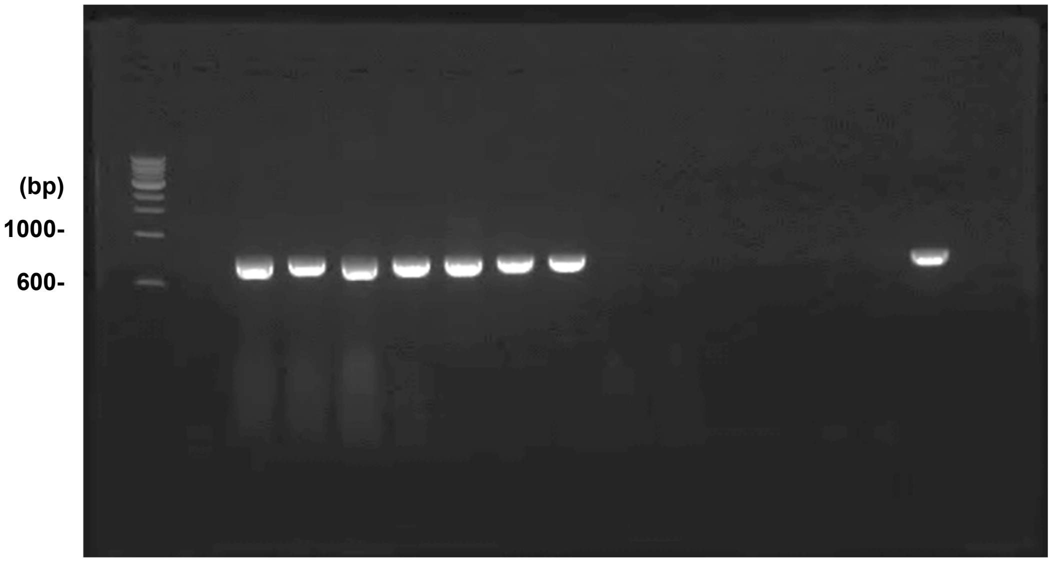

Figure 8A – Fungal DNA amplification using ITS primers

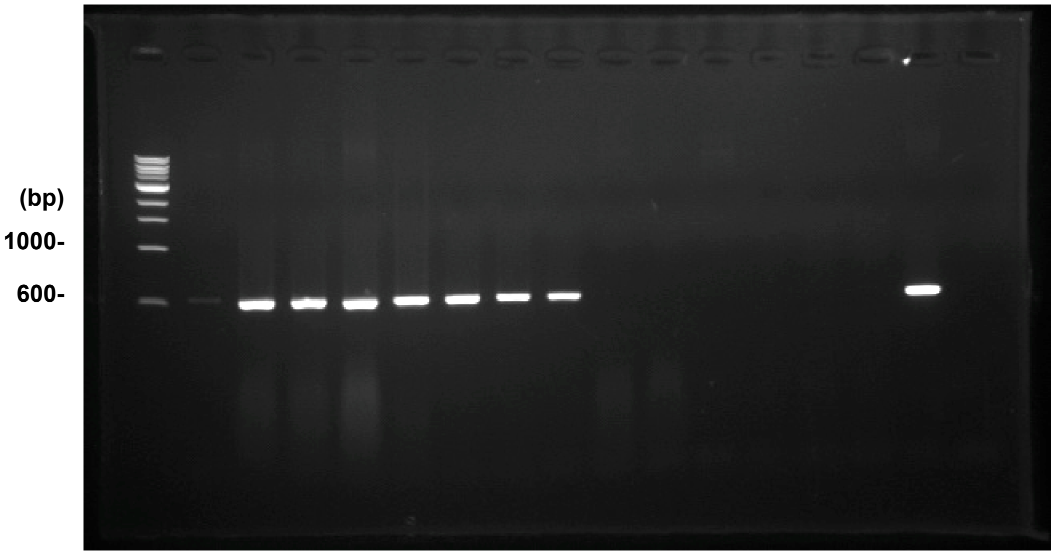

Figure 8B – Fungal DNA amplification using 28S rRNA primers

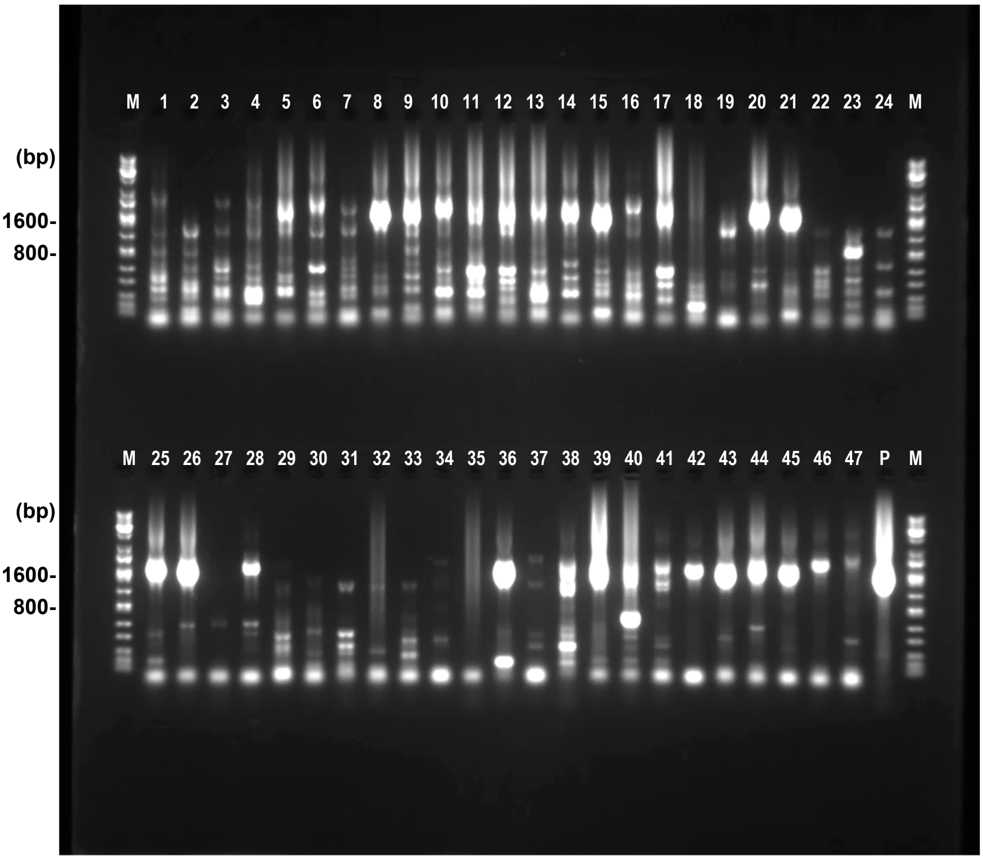

Detection of phytoplasma using universal primers targeting 16S rRNA on 9-month old symptomatic cassava samples (Sample 1-47; P - Positive Control *Ca.* P. luffae infected bitter gourd)

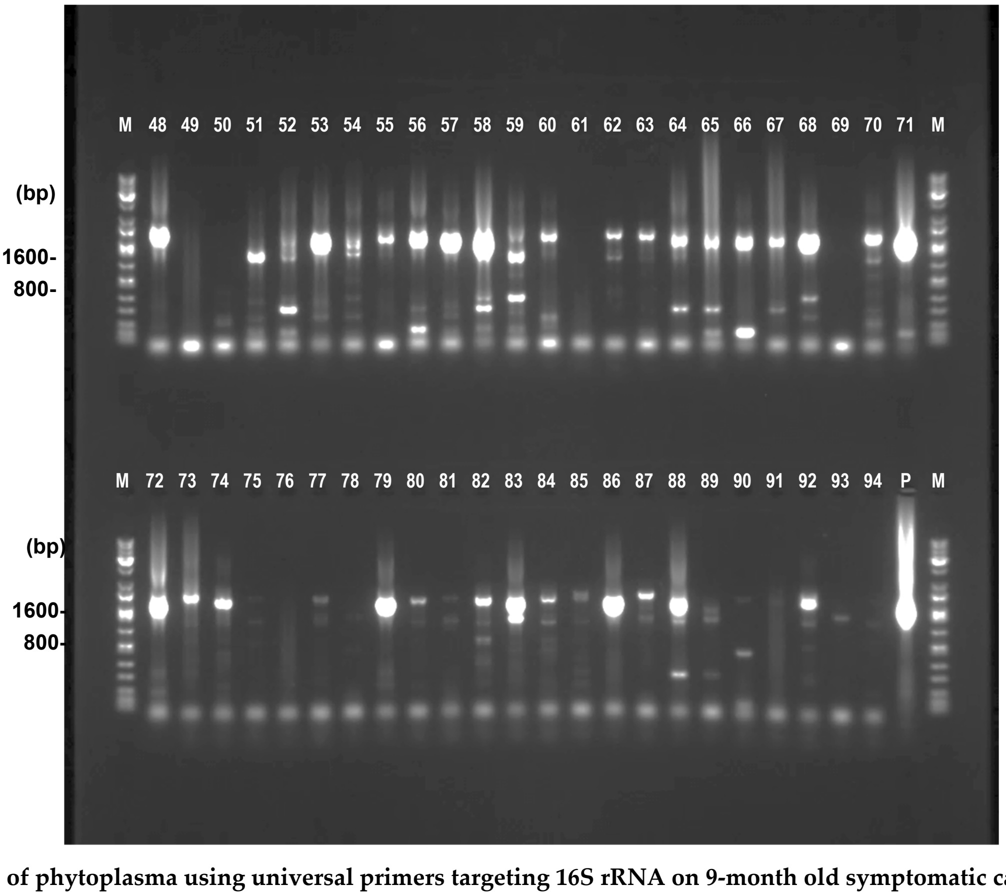

Detection of phytoplasma using universal primers targeting 16S rRNA on 9-month old symptomatic cassava samples (Sample 48-94; P - Positive Control *Ca.* P. luffae infected bitter gourd)

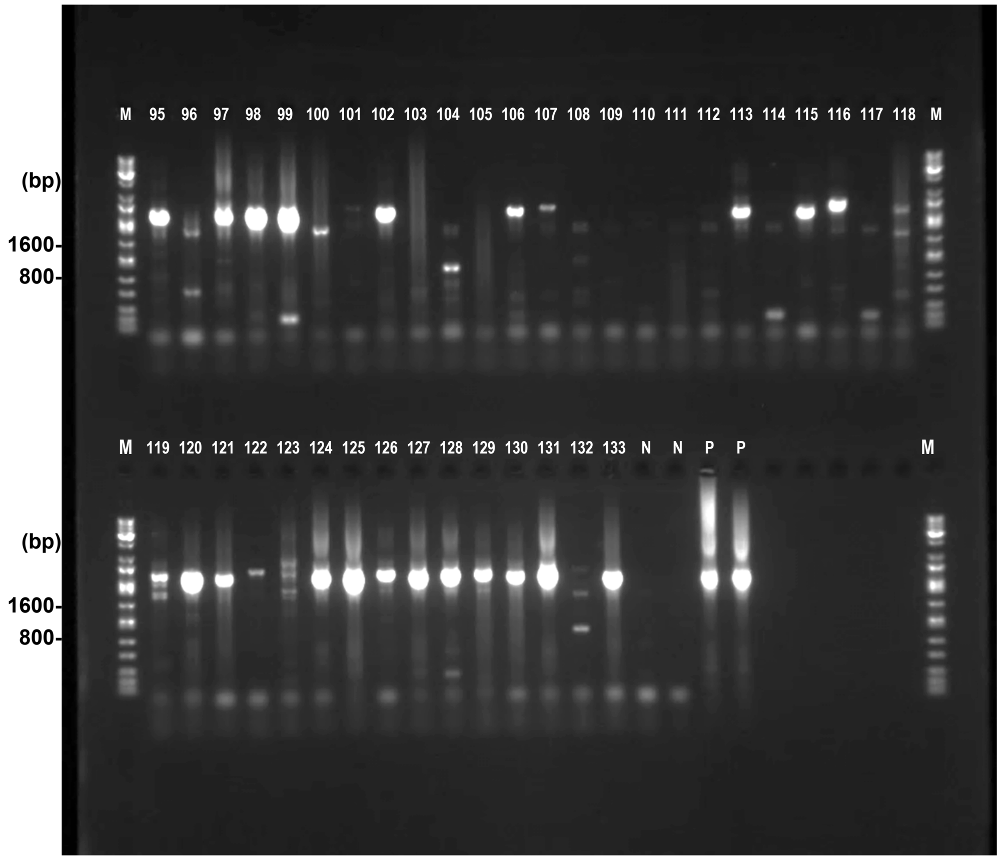

Detection of phytoplasma using universal primers targeting 16S rRNA on 9-month old symptomatic cassava samples (Sample 95-133; Controls: N – No template control; P - Positive Control *Ca.* P. luffae infected bitter gourd)

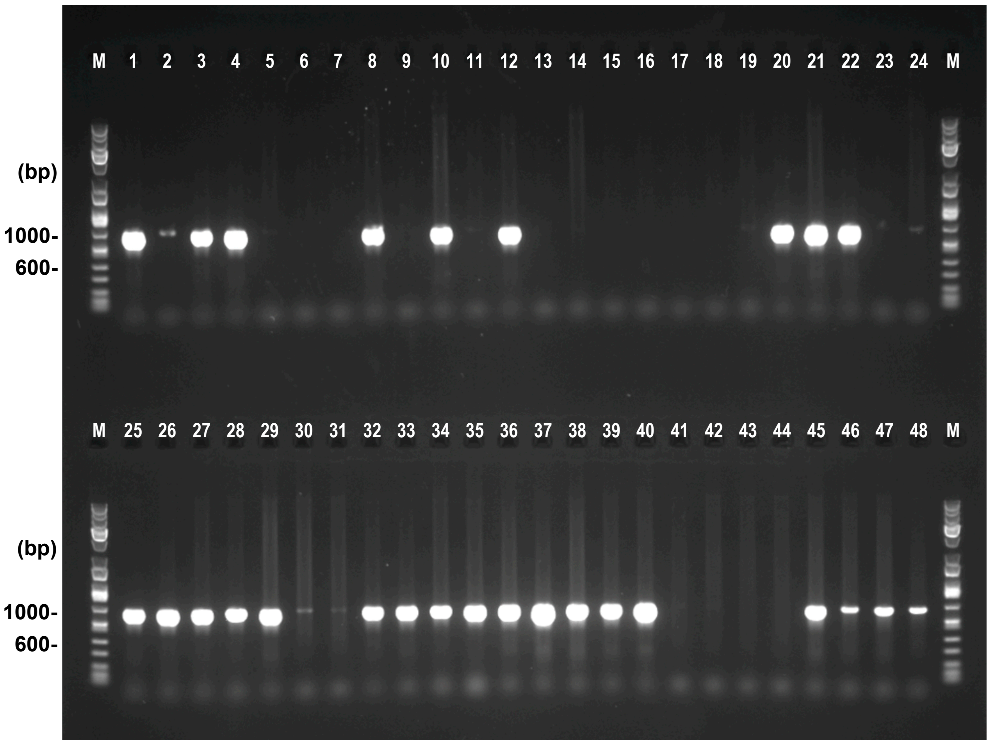

Detection of *C. theobromae* using specific primer targeting 28S rRNA on 7-month old symptomatic cassava samples (Sample 1-48)

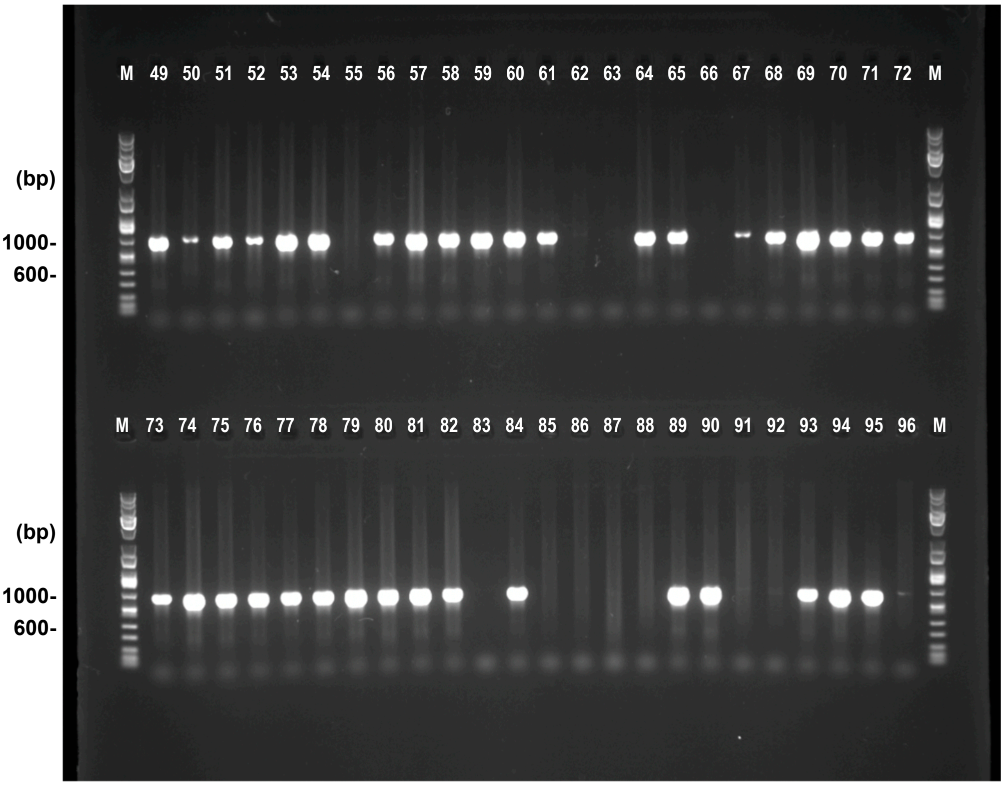

Detection of *C. theobromae* using specific primer targeting 28S rRNA on 7-month old symptomatic cassava samples (Sample 49-96)

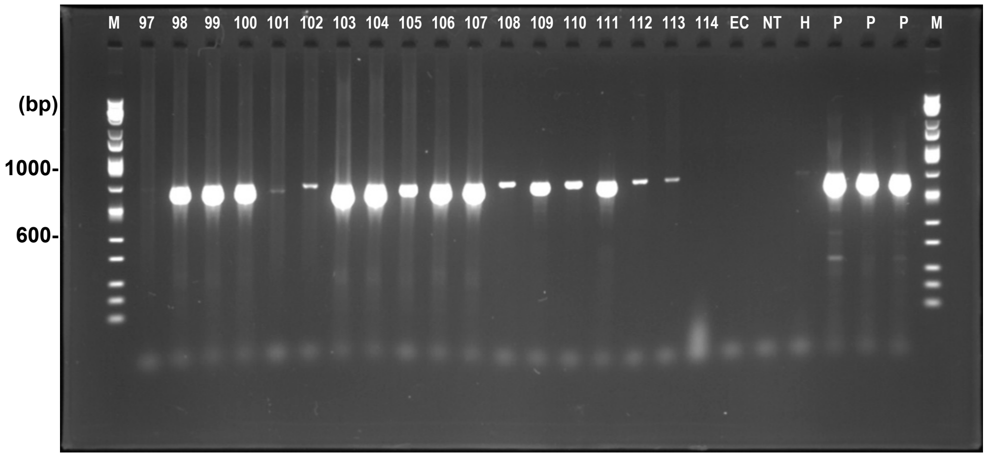

Detection of *C. theobromae* using specific primer targeting 28S rRNA on 7-month old symptomatic cassava samples (Sample 97-144; Controls: EC – Extraction Control; NT – No template control ; H – Asymptomatic cassava; P – Symptomatic cassava;

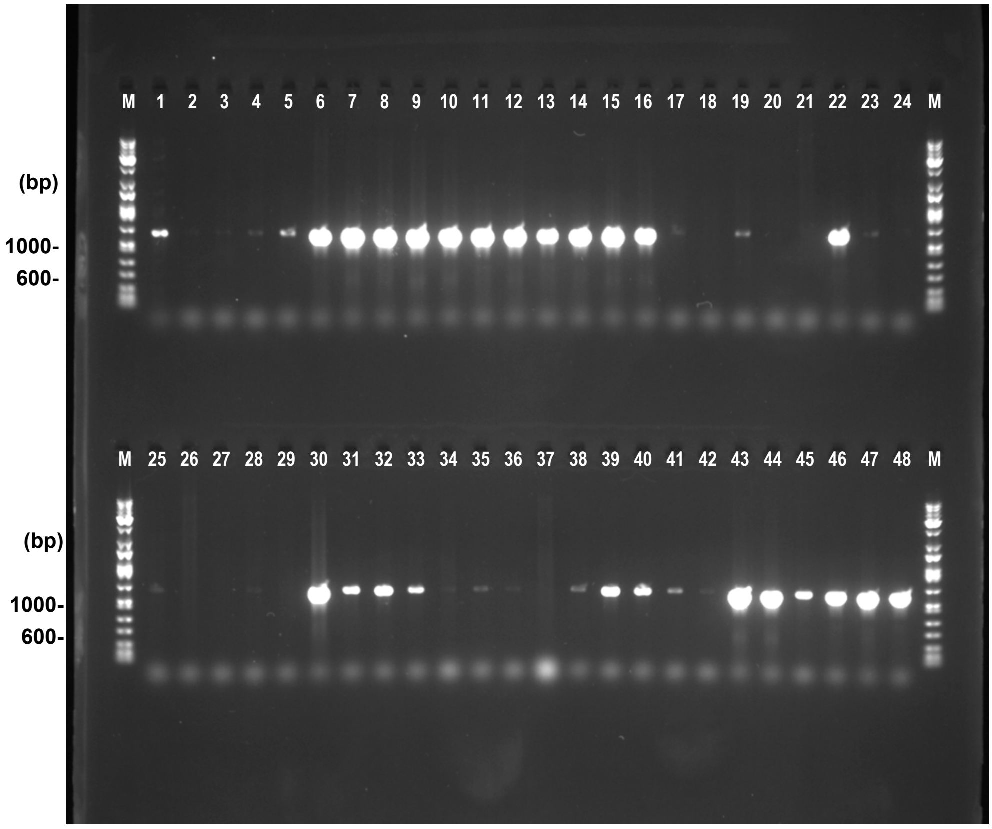

Detection of *C. theobromae* using specific primer targeting 28S rRNA on 9-month old symptomatic cassava samples (Sample 1-48)

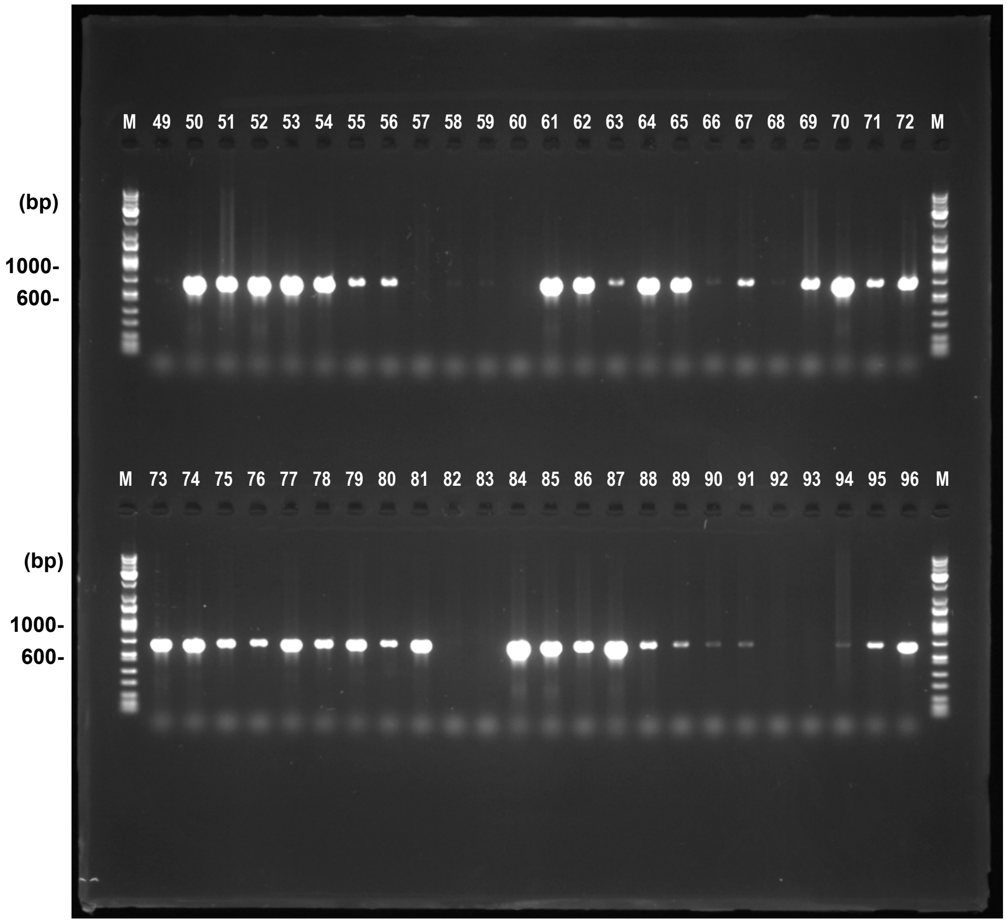

Detection of *C. theobromae* using specific primer targeting 28S rRNA on 9-month old symptomatic cassava samples (Sample 49-96)

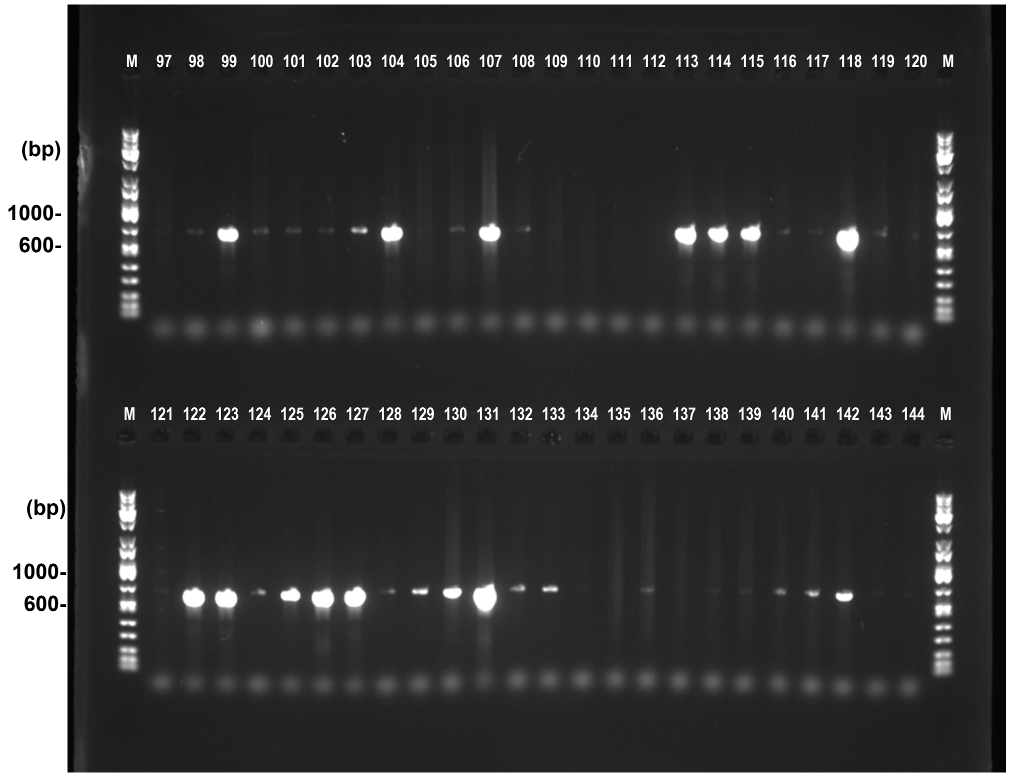

Detection of *C. theobromae* using specific primer targeting 28S rRNA on 9-month old symptomatic cassava samples (Sample 97-144)

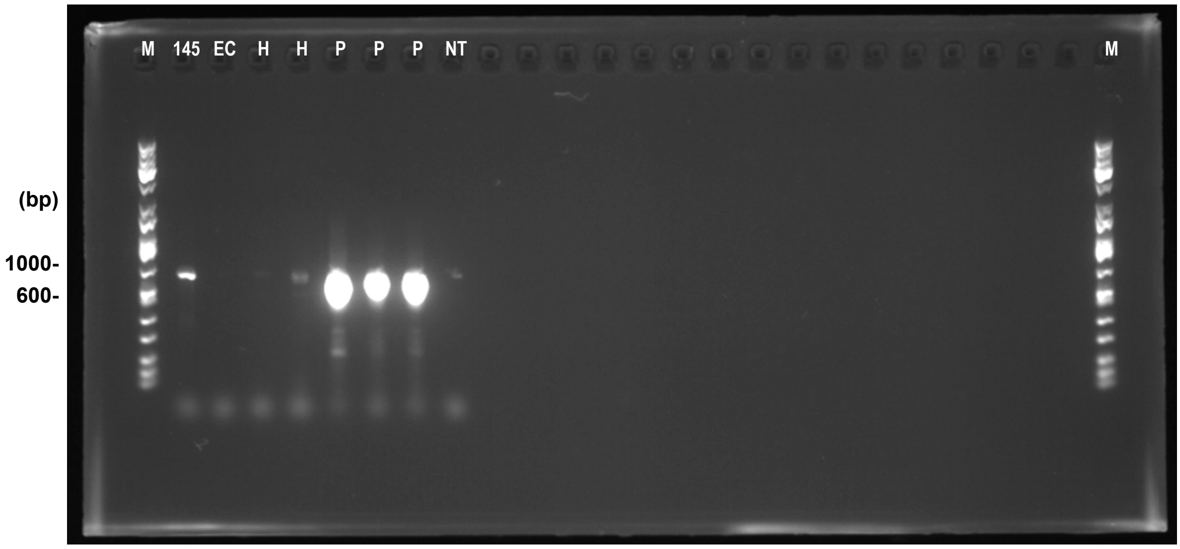

Detection of *C. theobromae* using specific primer targeting 28S rRNA on 9-month old symptomatic cassava samples (Sample 145-Controls: EC – Extraction control; H – Asymptomatic cassava; P – Symptomatic cassava; NT – No template control)

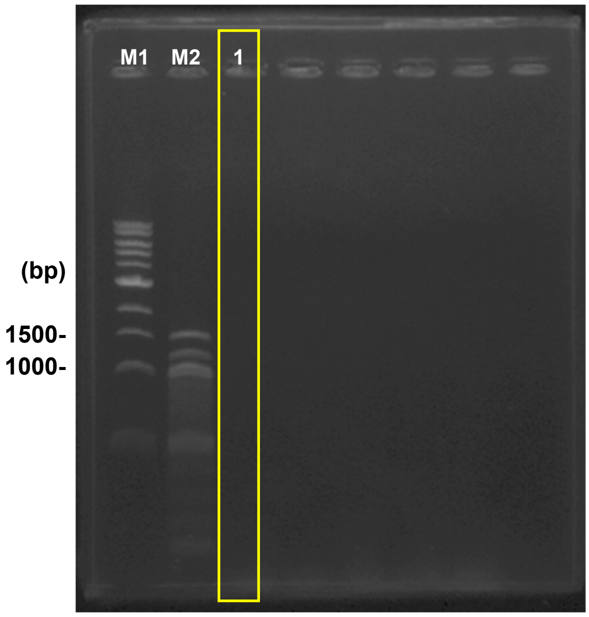

Figure 8B: Detection of *C. theobromae* using specific primer targeting 28S rRNA (phytoplasma positive asymptomatic cassava from Japan)

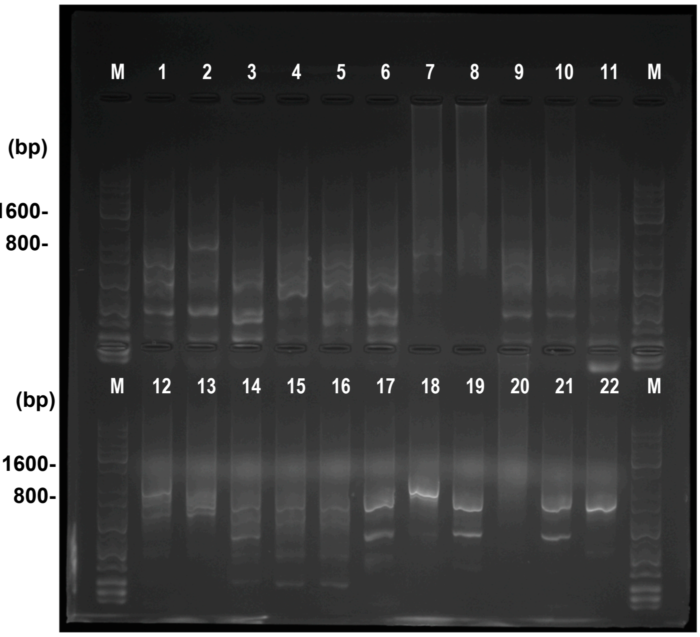

Detection of phytoplasma using universal primers targeting 16S rRNA on 9-month old asymptomatic cassava samples (Sample 1-22)

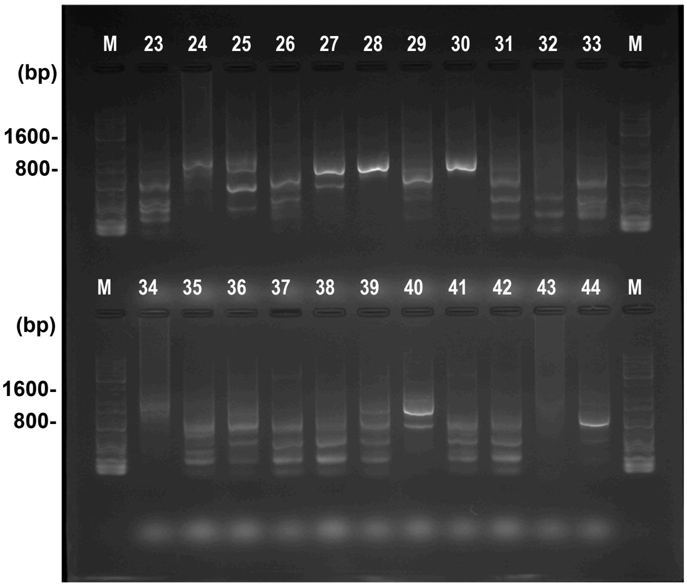

Detection of phytoplasma using universal primers targeting 16S rRNA on 9-month old asymptomatic cassava samples (Sample 23-44)

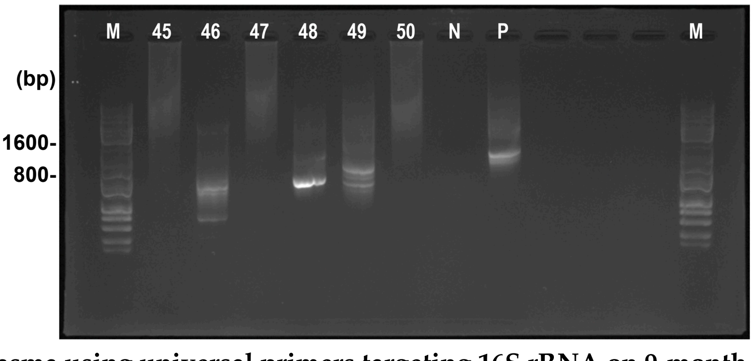

Detection of phytoplasma using universal primers targeting 16S rRNA on 9-month old asymptomatic cassava samples (Sample 45-50; N – No template control; P - Positive Control *Ca.* P. luffae infected bitter gourd)

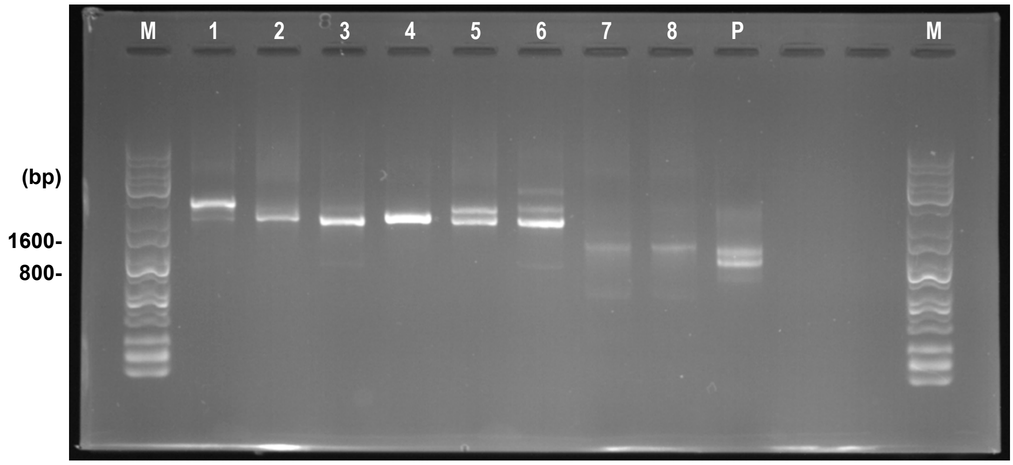

Restriction enzyme digestion of nested PCR products from 9-month old asymptomatic cassava using *Sca*I (Lanes 1-8; P – Positive Control *Ca.* P. luffae infected bitter gourd)

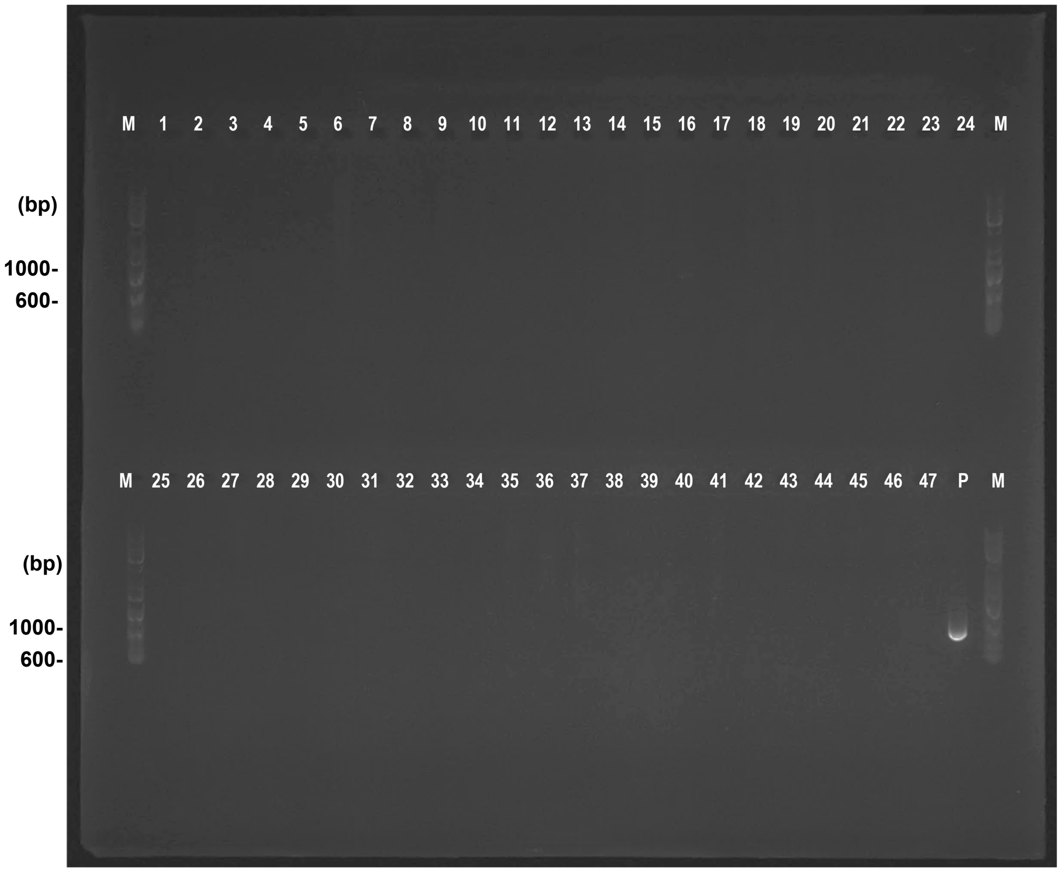

Detection of *C. theobromae* using specific primer targeting 28S rRNA on 9-month old asymptomatic cassava samples (Sample 1-47; P – CWBD-symptomatic cassava)
