# Supplementary material for: Status of Cassava Witches’ Broom Disease in the Philippines and Identification of Potential Pathogens by Metagenomic Analysis
Source: Biology (Basel). 2024 Jul 15;13(7):522. doi: 10.3390/biology13070522 (PMC11273669; doi:10.3390/biology13070522)
Supplement: Supplementary file 1 [file biology-13-00522-s001.zip › Table S1-Primer sequences and PCR conditions.pdf]

Table S1. Primer sequences and PCR conditions

Target	Primer Name	Sequence	Tm (°C)	Product Length	PCR Components Final Concentration and Volume)	PCR Running Conditions	Annealing Temp.	Reference
Nested PCR								
16S/23S	P1/<br>P7	5′-AAGAGTTTGATCCTGGCTCAGGATT-3′<br>5′-CGTCCTTCATCGGCTCTT-3′	58.1<br>54.6	1,800 bp	PCR buffer (invt) 1x<br>MgCl2 (kapa) 1.5 mM<br>dNTPs (invt) 0.2 mM<br>P1 (0.4 μM)<br>P7 (0.4 μM)<br>Taq (Kapa) 1.25 U/ μL<br>DNA template 1-2 μL<br>Nuclease-free water<br>Total Volume = 25 μL	Initial denaturation 94°C 2 mins<br>Denaturation 94°C 1 min<br>Annealing [**60 and 53°**C] 2 mins 35 cycles<br>Extension 72°C 1 min<br>Final Extension 72°C 10 mins	48°C -53°C, ±1	[1]
16S	R16mF2n/<br>R16mR1	5′-CATGCAAGTCGAACGGA-3′<br>5′-CTTAACCCCAATCATCGAC-3′	52.6<br>50.5	1,400 bp	PCR buffer (invt) 1x<br>MgCl2 (kapa) 1.5 mM<br>dNTPs (invt) 0.2 mM<br>R16mF2n (0.4 μM)<br>R16mR1 (0.4 μM)<br>Taq (Kapa) 1.25 U/μL<br>DNA template 1-2 μL<br>Nuclease-free water<br>Total Volume =25 μL	Initial denaturation 94°C 2 mins<br>Denaturation 94°C 1 min<br>Annealing [48°C] 2 mins 35 cycles<br>Extension 72°C 1 min<br>Final Extension 72°C 10 mins	48°C -53°C, ±1	[2,3]
Endpoint PCR								
ITS	ITS5/<br>ITS4	5′-GGAAGTAAAAGTCGT AACAAGG-3′<br>5′-TCCTCCGCTTATTGATATGC-3′	60.0<br>58.0	~700 bp	ExTaq PCR Mastermix 1x<br>dNTPs (Takara) 0.2 mM<br>ITS5 0.3 μM<br>ITS4 0.3 μM<br>DNA Template 1 μL<br>Taq (ExTaq) 1.5 U/μL<br>Nuclease-free water<br>Total Volume =25 μL	Initial denaturation 94°C 5 mins<br>Denaturation 94°C 1 min<br>Annealing [55°C] 30 sec 35 cycles<br>Extension 72°C 1 min<br>Final Extension 72°C 5 mins	N/A	[4]
28S	CTh_28SF/<br>CTh_28SR	5′-GAGCCTCCACCAGAGTTTCC-3′<br>5′-CTGGCAGTGTATTCTGTCCGA-3′	61.4<br>59.8	748 bp	PCR buffer (GoTaq) 2x<br>CTh_28SF (0.5 μM)<br>CTh_28SR (0.5 μM)<br>DNA template 1 μL<br>Nuclease-free water<br>Total Volume =10 μL	Initial denaturation 94°C 2 mins<br>Denaturation 94°C 30 sec<br>Annealing [55°C] 1 min 30 cycles<br>Extension 72°C 1 min<br>Final Extension 72°C 10 mins	N/A	This study

Amplicon sequencing								
V3V4 regions	341f/<br>805r	5′- ACACTCTTTCCCTACACGAGCTCTTCCGATCT-NNNNN-CCTACGGGNGGCWGCAG -3′<br><br>5′- GTGACTGGAGTTCAGAC-GTGTGCTCTTCCGATCTNNNNN-GAC-TACHVGGGTATCTAATCC -3′	N/A	300 bp	PCR Buffer (KOD FX Neo) 2×<br>dNTPs (each 2 mM)<br>341f (10 μM)<br>805r (10 μM)<br>DNA template 1 μL<br>KOD FX Neo (1.0 U/μL)	Initial denaturation 94℃ 2 min<br>Denaturation 98℃ 10 sec<br>Annealing 55℃ 30 sec X cycles<br>Extension 68℃ 30 sec<br>Final Extension 68℃ 7 min	N/A	[5,6]

References

1. Deng, S.; Hiruki, C. Amplification of 16S rRNA Genes from Culturable and Nonculturable Mollicutes. *J. Microbiol. Methods* **1991**, *14*, 53–61, doi:10.1016/0167-7012(91)90007-D.
2. Gundersen, D.E.; Lee, I.-M. Ultrasensitive Detection of Phytoplasmas by Nested-PCR Assays Using Two Universal Primer Pairs. *Phytopathol. Mediterr.* **1996**, *35*, 144–151.
3. Lee, I.-M. Universal Amplification and Analysis of Pathogen 16S rDNA for Classification and Identification of Mycoplasmalike Organisms. *Phytopathology* **1993**, *83*, 834–834, doi:10.1094/PHYTO-83-834.
4. White, T.J.; Bruns, T.; Lee, S.; Taylor, J. Amplification and Direct Sequencing of Fungal Ribosomal RNA Genes for Phylogenetics. In *PCR Protocols: A Guide to Methods and Applications*; Innis, M.A., Gelfand, D.H., Sninsky, J.J., White, T.J., Eds.; Academic Press: San Diego, 1990; pp. 315–322 ISBN 978-0-12-372180-8.
5. Gantner, S.; Andersson, A.F.; Alonso-Sáez, L.; Bertilsson, S. Novel Primers for 16S rRNA-Based Archaeal Community Analyses in Environmental Samples. *J. Microbiol. Methods* **2011**, *84*, 12–18, doi:10.1016/J.MIMET.2010.10.001.
6. Muyzer, G.; De Waal, E.C.; Uitterlinden, A.G. Profiling of Complex Microbial Populations by Denaturing Gradient Gel Electrophoresis Analysis of Polymerase Chain Reaction-Amplified Genes Coding for 16S rRNA. *Appl. Environ. Microbiol.* **1993**, *59*, 695–700, doi:10.1128/AEM.59.3.695-700.1993.
