# Supplementary material for: Status of Cassava Witches’ Broom Disease in the Philippines and Identification of Potential Pathogens by Metagenomic Analysis
Source: Biology (Basel). 2024 Jul 15;13(7):522. doi: 10.3390/biology13070522 (PMC11273669; doi:10.3390/biology13070522)
Supplement: Supplementary file 1 [file biology-13-00522-s001.zip › Table S2-Sequence accession numbers and BLAST result.pdf]

Table S2. Sequence accession numbers and BLAST result

Host	Target	Accession No.	Length (bp)	Sequence Name	BLAST Hit	% Identity	Bit Score	Classification[1]
Cassava	16S	OQ797676.1	1,131	Uncultured bacterium clone CV-Ub1 16S ribosomal RNA gene, partial sequence	FM872874.1. Uncultured bacterium partial 16S rRNA gene, clone FB04A12	100%	2006	Not phytoplasma
	16S	OQ797677.1	1,209	Uncultured Bacillus sp. clone CV-Ub2 16S ribosomal RNA gene, partial sequence	MT184818.1. Bacillus aryabhattai strain NWPZ-6 16S ribosomal RNA gene, partial sequence	99%	2222	Not phytoplasma
	16S	OQ797678.1	1,191	Uncultured Bacillus sp. clone CV-Ub3 16S ribosomal RNA gene, partial sequence	OQ560572.1. Priestia flexa strain CAD2 16S ribosomal RNA gene, partial sequence	100%	2200	Not phytoplasma
	16S	OQ797679.1	1,176	Uncultured Bacillus sp. clone CV-Ub4 16S ribosomal RNA gene, partial sequence	OQ560572.1. Priestia flexa strain CAD2 16S ribosomal RNA gene, partial sequence	100%	2167	Not phytoplasma
	16S	OQ797680.1	1,179	Uncultured Bacillus sp. clone CV-Ub5 16S ribosomal RNA gene, partial sequence	OQ553824.1. Priestia megaterium strain wy-22 16S ribosomal RNA gene, partial sequence	100%	2170	Not phytoplasma
	16S	OQ797681.1	1,311	Uncultured bacterium clone CV-Ub6 16S ribosomal RNA gene, partial sequence	KX035309. Uncultured Anaerolineaceae bacterium clone MZGKrs25 16S ribosomal RNA gene, partial sequence	98.7%	2327.9	Not phytoplasma
	16S	OQ797682.1	1,357	Uncultured bacterium clone CV-Ub7 16S ribosomal RNA gene, partial sequence	FR687440. Uncultured bacterium partial 16S rRNA gene, clone 500-36	99.0%	2427.62	Not phytoplasma
	16S	OQ797683.1	1,296	Uncultured bacterium clone CV-Ub8 16S ribosomal RNA gene, partial sequence	JN409204. Uncultured Chloroflexi bacterium clone HG-B02125 16S ribosomal RNA gene, partial sequence	99.2%	2337.13	Not phytoplasma
	16S	OQ797684.1	1,357	Uncultured bacterium clone CV-Ub9 16S ribosomal RNA gene, partial sequence	GQ179679. Uncultured Pelosinus sp. clone VE44F01 16S ribosomal RNA gene, partial sequence	99.4%	2460.86	Not phytoplasma
	16S	OQ797685.1	1,458	Uncultured bacterium clone CV-Ub10 16S ribosomal RNA gene, partial sequence	NR_117667. Sporomusa sphaeroides DSM 2875 16S ribosomal RNA, partial sequence	94.7%	2128.46	Not phytoplasma
	16S	OQ797686.1	1,050	Uncultured bacterium clone CV-Ub11 16S ribosomal RNA gene, partial sequence	NR_153708. Saccharibacillus endophyticus strain JM-1350 16S ribosomal RNA, partial sequence	99.7%	2008.43	Not phytoplasma
	16S	OQ797687.1	1,333	*Candidatus* Phytoplasma luffae clone CV-Phy1 16S ribosomal RNA gene, partial sequence	MN784933.1. *Candidatus* Phytoplasma luffae clone CWB17a 16S ribosomal RNA gene, partial sequence	99.70%	2440	16SrVIII-A
	16S	OQ797688.1	1,322	*Candidatus* Phytoplasma luffae clone CV-Phy2 16S ribosomal RNA gene, partial sequence	MN784933.1. *Candidatus* Phytoplasma luffae clone CWB17a 16S ribosomal RNA gene, partial sequence	99.85%	2431	16SrVIII-A
	16S	OR673512	1,289	*Candidatus* Phytoplasma luffae clone CV-Phy3 16S ribosomal RNA gene, partial sequence	MW403979. *Candidatus* Phytoplasma luffae clone PT1 16S ribosomal RNA gene and 16S-23S ribosomal RNA intergenic spacer, partial sequence.	99.9%	2374.07	16SrVIII-A

	16S	OR673513	1,289	*Candidatus* Phytoplasma luffae clone CV-Phy4 16S ribosomal RNA gene, partial sequence	MW403979. *Candidatus* Phytoplasma luffae clone PT1 16S ribosomal RNA gene and 16S-23S ribosomal RNA intergenic spacer, partial sequence.	99.8%	2368.53	16SrVIII-A
	16S	OR673511	1,289	*Candidatus* Phytoplasma luffae clone CV-Phy5 16S ribosomal RNA gene, partial sequence	MW403979. *Candidatus* Phytoplasma luffae clone PT1 16S ribosomal RNA gene and 16S-23S ribosomal RNA intergenic spacer, partial sequence.	99.8%	2368.53	16SrVIII-A
	16S	OR673514	1,289	*Candidatus* Phytoplasma luffae clone CV-Phy6 16S ribosomal RNA gene, partial sequence	MW403979. *Candidatus* Phytoplasma luffae clone PT1 16S ribosomal RNA gene and 16S-23S ribosomal RNA intergenic spacer, partial sequence.	99.9%	2374.07	16SrVIII-A
	16S	OQ861100.1	1394	*Candidatus* Phytoplasma luffae clone CWB24 16S ribosomal RNA gene, partial sequence	MN784937.1. *Candidatus* Phytoplasma luffae clone CWB28 16S ribosomal RNA gene, partial sequence	99.71%	2531	16SrVIII-A
	16S	OQ861101.1	1451	*Candidatus* Phytoplasma luffae clone CWB29 16S ribosomal RNA gene, partial sequence	MN784934.1. *Candidatus* Phytoplasma luffae clone CWB17b 16S ribosomal RNA gene, partial sequence	99.16%	2582	16SrVIII-A
	ITS	OR415096.1	696	*Ceratobasidium theobromae* clone CV-Cth1 small subunit ribosomal RNA gene, partial sequence; internal transcribed spacer 1, 5.8S ribosomal RNA gene, and internal transcribed spacer 2, complete sequence; and large subunit ribosomal RNA gene, partial sequence	KU255724. *Ceratobasidium theobromae* strain South Sulawesi 2 18S ribosomal RNA gene, partial sequence; internal transcribed spacer 1, 5.8S ribosomal RNA gene, and internal transcribed spacer 2, complete sequence; and 28S ribosomal RNA gene, partial sequence	100%	1286.39	*Ceratobasidium theobromae*
	ITS	OR415097.1	697	*Ceratobasidium theobromae* clone CV-Cth2 small subunit ribosomal RNA gene, partial sequence; internal transcribed spacer 1, 5.8S ribosomal RNA gene, and internal transcribed spacer 2, complete sequence; and large subunit ribosomal RNA gene, partial sequence	KU255724. *Ceratobasidium theobromae* strain South Sulawesi 2 18S ribosomal RNA gene, partial sequence; internal transcribed spacer 1, 5.8S ribosomal RNA gene, and internal transcribed spacer 2, complete sequence; and 28S ribosomal RNA gene, partial sequence	100%	1288.24	*Ceratobasidium theobromae*
	ITS	OR415098.1	696	*Ceratobasidium theobromae* clone CV-Cth3 small subunit ribosomal RNA gene, partial sequence; internal transcribed spacer 1, 5.8S ribosomal RNA gene, and internal transcribed spacer 2, complete sequence; and large subunit ribosomal RNA gene, partial sequence	KU255724. *Ceratobasidium theobromae* strain South Sulawesi 2 18S ribosomal RNA gene, partial sequence; internal transcribed spacer 1, 5.8S ribosomal RNA gene, and internal transcribed spacer 2, complete sequence; and 28S ribosomal RNA gene, partial sequence	100%	1286.39	*Ceratobasidium theobromae*
	ITS	OR415099.1	699	*Ceratobasidium theobromae* clone CV-Cth4 small subunit ribosomal RNA gene, partial sequence; internal transcribed spacer 1, 5.8S ribosomal RNA	KU255724. *Ceratobasidium theobromae* strain South Sulawesi 2 18S ribosomal RNA gene, partial sequence; internal transcribed spacer 1,	100%	1291.93	*Ceratobasidium theobromae*

			gene, and internal transcribed spacer 2, complete sequence; and large subunit ribosomal RNA gene, partial sequence	5.8S ribosomal RNA gene, and internal transcribed spacer 2, complete sequence; and 28S ribosomal RNA gene, partial sequence			
ITS	OR415100.1	702	*Ceratobasidium theobromae* clone CV-Cth5 small subunit ribosomal RNAgene, partial sequence; internal transcribed spacer 1, 5.8S ribosomal RNA gene, and internal transcribed spacer 2, complete sequence; and large subunit ribosomal RNA gene, partial sequence.	KU255724. *Ceratobasidium theobromae* strain South Sulawesi 2 18S ribosomal RNA gene, partial sequence; internal transcribed spacer 1, 5.8S ribosomal RNA gene, and internal transcribed spacer 2, complete sequence; and 28S ribosomal RNA gene, partial sequence	100%	1297.47	*Ceratobasidium theobromae*
ITS	OR415101.1	596	*Ceratobasidium theobromae* clone CV-Cth6 small subunit ribosomal RNAgene, partial sequence; internal transcribed spacer 1, 5.8S ribosomal RNA gene, and internal transcribed spacer 2, complete sequence; and large subunit ribosomal RNA gene, partial sequence.	KU255724. Ceratobasidium theobromae strain South Sulawesi 2 18S ribosomal RNA gene, partial sequence; internal transcribed spacer 1, 5.8S ribosomal RNA gene, and internal transcribed spacer 2, complete sequence; and 28S ribosomal RNA gene, partial sequence	100%	1101.72	*Ceratobasidium theobromae*
28S	OR673502	714	*Ceratobasidium theobromae* clone CV-Cth7 large subunit ribosomal RNA gene, partial sequence	OQ361384. *Ceratobasidium theobromae* isolate 22VDACS-RT12 large subunit ribosomal RNA gene, partial sequence	100.0%	1319.63	*Ceratobasidium theobromae*
28S	OR673503	715	*Ceratobasidium theobromae* clone CV-Cth8 large subunit ribosomal RNA gene, partial sequence	OQ361384. *Ceratobasidium theobromae* isolate 22VDACS-RT12 large subunit ribosomal RNA gene, partial sequence	100.0%	1321.47	*Ceratobasidium theobromae*
28S	OR673504	720	Ceratobasidium theobromae clone CV-Cth9 large subunit ribosomal RNA gene, partial sequence	OQ361384. *Ceratobasidium theobromae* isolate 22VDACS-RT12 large subunit ribosomal RNA gene, partial sequence	100.0%	1330.71	*Ceratobasidium theobromae*
28S	OR673505	715	*Ceratobasidium theobromae* clone CV-Cth10 large subunit ribosomal RNA gene, partial sequence	OQ361384. *Ceratobasidium theobromae* isolate 22VDACS-RT12 large subunit ribosomal RNA gene, partial sequence	100.0%	1321.47	*Ceratobasidium theobromae*
28S	OR673506	717	*Ceratobasidium theobromae* clone CV-Cth11 large subunit ribosomal RNA gene, partial sequence	OQ361384. *Ceratobasidium theobromae* isolate 22VDACS-RT12 large subunit ribosomal RNA gene, partial sequence	100.0%	1325.17	*Ceratobasidium theobromae*
28S	OR673507	717	*Ceratobasidium theobromae* clone CV-Cth12 large subunit ribosomal RNA gene, partial sequence	OQ361384. *Ceratobasidium theobromae* isolate 22VDACS-RT12 large subunit ribosomal RNA gene, partial sequence	100.0%	1325.17	*Ceratobasidium theobromae*
28S	OR673508	715	*Ceratobasidium theobromae* clone CV-Cth13 large subunit ribosomal RNA gene, partial sequence	OQ361384. *Ceratobasidium theobromae* isolate 22VDACS-RT12 large subunit ribosomal RNA gene, partial sequence	99.9%	1315.93	*Ceratobasidium theobromae*

[1]Based on *iPhyClassifier* (16S)
