# Supplementary material for: Status of Cassava Witches’ Broom Disease in the Philippines and Identification of Potential Pathogens by Metagenomic Analysis
Source: Biology (Basel). 2024 Jul 15;13(7):522. doi: 10.3390/biology13070522 (PMC11273669; doi:10.3390/biology13070522)
Supplement: Supplementary file 1 [file biology-13-00522-s001.zip › Table S3-Summary of samples used for NGS analysis.pdf]

Table S3. Summary of samples used for NGS analysis

Sample Name	Age	Location	Sample Condition	Tissue type	Method Used	SRA Accession Number
A-1	3 months	Bukidnon	Symptomatic	Leaves (Field collected)	16S amplicon sequencing (PRJNA956085)	SRR24182386
A-2	3 months	Bukidnon	Symptomatic	Leaves (Field collected)	16S amplicon sequencing (PRJNA956085)	SRR24182385
A-3	8 months	Bukidnon	Symptomatic	Leaves (Field collected)	16S amplicon sequencing (PRJNA956085)	SRR24182384
A-3	8 months	Bukidnon	Symptomatic	Leaves (Field collected)	16S amplicon sequencing (PRJNA956085)	SRR24182383
B1	7 months	Isabela	Symptomatic	Leaves (Field collected)	16S amplicon sequencing (PRJNA956085)	SRR24182382
B2	7 months	Isabela	Symptomatic	Leaves (Field collected)	16S amplicon sequencing (PRJNA956085)	SRR24182381
CV-A	More than 3 months (maturity stage)	Bukidnon	Symptomatic	Leaves (Field collected)	Shotgun sequencing (PRJNA1003250)	SRR25604658
CV-B	7 months	Isabela	Symptomatic	Leaves (Field collected)	Shotgun sequencing (PRJNA1003250)	SRR25604657
I-A	1 month	Bukidnon	Symptomatic	Leaves (Screenhouse propageted)	RNA Seq (PRJNA990661)	SRR25113839
I-B	1 month	Isabela	Symptomatic	Leaves (Screenhouse propageted)	RNA Seq (PRJNA990661)	SRR25113838
I-1	3 months	Isabela	Symptomatic	Leaves (Field collected)	RNA Seq (PRJNA1028178)	SRR26387394
I-2	3 months	Isabela	Symptomatic	Leaves (Field collected)	RNA Seq (PRJNA1028178)	SRR26387393
I-3	3 months	Isabela	Symptomatic	Leaves (Field collected)	RNA Seq (PRJNA1028178)	SRR26387392
H-1	3 months	Isabela	Asymptomatic	Leaves (Field collected)	RNA Seq (PRJNA1028178)	SRR26387397
H-2	3 months	Isabela	Asymptomatic	Leaves (Field collected)	RNA Seq (PRJNA1028178)	SRR26387396
H-3	3 months	Isabela	Asymptomatic	Leaves (Field collected)	RNA Seq (PRJNA1028178)	SRR26387395
