# Supplementary material for: Status of Cassava Witches’ Broom Disease in the Philippines and Identification of Potential Pathogens by Metagenomic Analysis
Source: Biology (Basel). 2024 Jul 15;13(7):522. doi: 10.3390/biology13070522 (PMC11273669; doi:10.3390/biology13070522)
Supplement: Supplementary file 1 [file biology-13-00522-s001.zip › Table S4-Result of host-read filtering using HISAT2.pdf]

Table S4. Result of host-read filtering using HISAT2

Sample Name	No. total reads (pairs)	No. unmapped reads	Over-all alignment rate to host genome
DNA			
CV-A	104,340,074	33,162,614	73.75%
CV-B	100,800,674	21,955,029	82.62%
RNA			
I-A	121,577,770	12,284,237	82.13%
I-B	130,153,010	8,317,472	91.08%
I-1	18,206,192	2,343,397	89.41%
I-2	17,922,178	2,312,619	89.47%
I-3	15,968,431	1,911,701	90.16%
H-1	16,908,658	1,367,970	94.73%
H-2	12,932,045	1,606,176	90.84%
H-3	16,817,590	1,198,332	95.32%

[1]Aligned concordantly or discordantly 0 time
