# Supplementary material for: Status of Cassava Witches’ Broom Disease in the Philippines and Identification of Potential Pathogens by Metagenomic Analysis
Source: Biology (Basel). 2024 Jul 15;13(7):522. doi: 10.3390/biology13070522 (PMC11273669; doi:10.3390/biology13070522)
Supplement: Supplementary file 1 [file biology-13-00522-s001.zip › Table S5-Genomic and transcriptomic reads classification.pdf]

Table S5. Genomic and transcriptomic reads classification.

Classification	No. reads	
	**CV-A**	**CV-B**
No. of reads	**27,812,716**	**27,586,067**
Bacteria	23,786,049 (85.53%)	23,642,917 (85.71%)
Fungi	1,558,170 (5.61%)	795,074 (2.89%)
Viruses	77,152 (0.28%)	85,775 (0.32%)
Archaea	5,446 (0.02%)	4,603 (0.02%)
other microbes	2,370,147 (8.53%)	3,046,511 (11.05%)
unclassified	15,752 (0.06%)	11,187 (0.05%)
Total classified	27,796,964 (99.94%)	27,574,880 (99.96%)

Classification	No. contigs	
	CV-A	CV-B
No. of contigs	309,964	199,721
Bacteria	107,369 (91.61%)	33,008 (66.62%)
Fungi	8,436 (7.2%)	15,592 (31.47%)
Viruses	120 (0.10%)	42 (0.08%)
Archaea	28 (0.02%)	11 (0.02%)
other microbes	1,115 (0.95%)	878 (1.77%)
unclassified	129 (0.11%)	18 (0.04%)
Total Classified	117,068 (99.89%)	49,531 (99.96%)
Total contigs analyzed	117,197	49,549

	Symptomatic					Asymptomatic		
Stage	1-month-old[1]		3-month-old[2]					
Classification	I-A	I-B	I-1	I-2	I-3	H-1	H-2	H-3
Fungi	868,567 (40.8%)	113,203 (10.6%)	564,656 (85.3%)	357,067 (81.8%)	288,534 (82.9%)	17,056 (11.7%)	11,098 (4.6%)	7,590 (7.0%)
Bacteria	775,953 (36.3%)	649,919 (60.5%)	58,982 (8.9%)	49,850 (11.4%)	36,943 (10.6%)	84,960 (58.1%)	177,692 (73.5%)	62,295 (57.9%)
Viruses	363,98 (1.7%)	2,367 (0.2%)	3,980 (0.6%)	3,204 (0.7%)	4,847 (1.4%)	605 (0.4%)	796 (0.3%)	492 (0.5%)
Archaea	1,788 (0.1%)	746 (0.1%)	383 (0.1%)	304 (0.1%)	206 (0.1%)	384 (0.3%)	1,203 (0.5%)	296 (0.3%)
Others	446,782 (20.9%)	305,219 (28.4%)	33,124 (5.0%)	25,668 (5.9%)	17,116 (4.9%)	42,699 (29.2%)	50,174 (20.8%)	36,642 (34%)

Unclassified	8,718 (0.4%)	2,354 (0.2%)	503 (0.1%)	350 (0.1%)	307 (0.1%)	523 (0.4%)	670 (0.3%)	351 (0.3%)
Total classified	2,129,488 (99.6%)	1,071,454 (99.8%)	661,125 (99.9%)	436,093 (99.9%)	347,646 (99.9%)	145,704 (99.6%)	240,963 (99.7%)	107,315 (99.7%)
**Total reads**	**2,138,206**	**1,073,808**	**661,628**	**436,443**	**347,953**	**146,227**	**241,633**	**107,666**

[1]Leaves collected from screenhouse-propagated 1-month-old CWBD symptomatic cassava from Bukidnon (I-A) and Isabela (I-B)

[2]Field-collected leaves from 3-month-old CWBD symptomatic cassava in Isabela
