# Supplementary material for: Status of Cassava Witches’ Broom Disease in the Philippines and Identification of Potential Pathogens by Metagenomic Analysis
Source: Biology (Basel). 2024 Jul 15;13(7):522. doi: 10.3390/biology13070522 (PMC11273669; doi:10.3390/biology13070522)
Supplement: Supplementary file 1 [file biology-13-00522-s001.zip › Table S6-Detection of phytoplasma and Ceratobasidium in individual plants.pdf]

Table S6. Detection of phytoplasma and *Ceratobasidium* in individual plants

Plant Name	7 months			9 months				
	16S Nested PCR		*Ceratobasidium*	16S Nested PCR				*Ceratobasidium*
	leaves	*Sca*I	leaves	leaves	roots	stem	*Sca*I	leaves
Symptomatic								
A	0/2	NT[1]	0/2	NT[1]	0/1	2/2	0/2	0/2
B	1/5	0/1	1/5	0/2	1/1	4/7	0/5	1/5
C	3/13	0/3	3/13	1/1	0/1	4/7	0/5	3/13
D	4/6	0/4	4/6	0/1	NT	3/8	0/3	4/6
E	4/7	0/4	4/7	NT[1]	0/1	0/2	NT[1]	4/7
F	2/6	0/2	2/6	0/2	0/1	3/4	1/3	2/6
G	5/6	0/5	5/6	0/3	0/1	0/7	NT[1]	5/6
H	4/7	0/4	4/7	1/1	1/1	4/6	0/6	4/7
I	7/7	0/7	7/7	2/2	1/1	3/7	0/6	7/7
J	3/4	0/3	3/4	NT[1]	0/1	0/4	NT[1]	3/4
K	1/6	0/1	1/6	0/2	1/1	3/7	0/4	1/6
L	2/8	0/2	2/8	0/1	0/1	3/8	0/3	2/8
M	2/7	0/2	2/7	2/4	0/1	4/9	1/6	2/7
N	4/7	0/4	4/7	0/1	0/1	2/9	0/2	4/7
O	0/2	NT	0/2	0/1	0/1	1/2	0/1	0/2
P	4/6	0/4	4/6	2/2	0/1	4/8	0/6	4/6
Q	3/6	0/3	3/6	NT[1]	NT[1]	NT[1]	NT[1]	3/6
Asymptomatic	NT[1]	NT[1]	NT[1]	9/50	NT[1]	NT[1]	0/9	0/50

[1]Not tested
