# Supplementary figures and images for: Status of Cassava Witches’ Broom Disease in the Philippines and Identification of Potential Pathogens by Metagenomic Analysis
Source: Biology (Basel). 2024 Jul 15;13(7):522. doi: 10.3390/biology13070522 (PMC11273669; doi:10.3390/biology13070522)

**Figure S2.** *In-silico* ScaI restriction enzyme digestion of 16S rDNA sequences

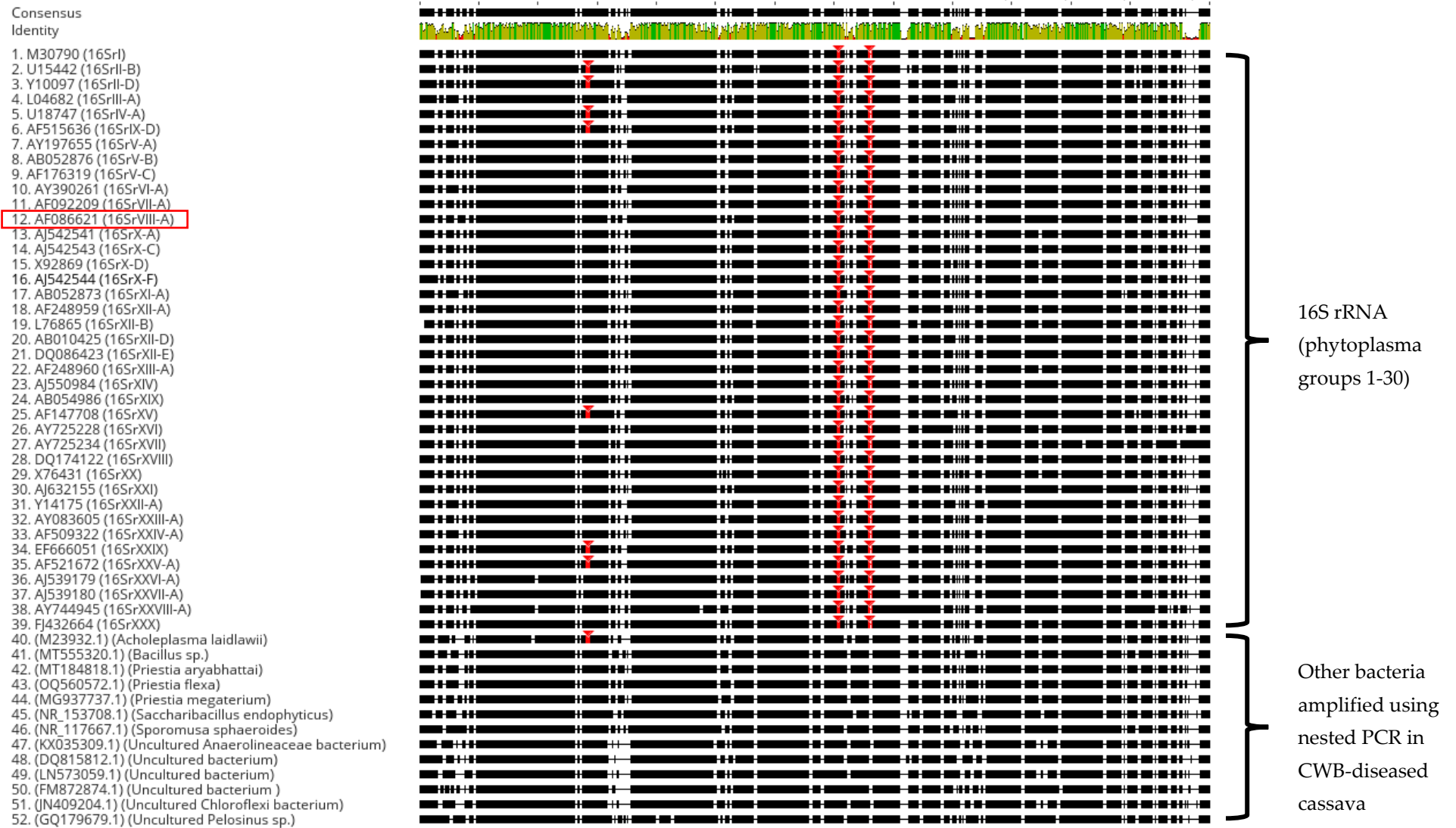

Supplement: Supplementary file 1 [file biology-13-00522-s001.zip › Figure S2-In-silico ScaI restriction enzyme digestion of 16S rDNA sequences.pdf]
